# Slack reactants: A state-space truncation framework to estimate quantitative behavior of the chemical master equation

**DOI:** 10.1063/5.0013457

**Published:** 2020-08-05

**Authors:** Jinsu Kim, Jason Dark, German Enciso, Suzanne Sindi

**Affiliations:** 1 Department of Mathematics, University of California, Irvine, California 92697, USA; 2 Department of Applied Mathematics, University of California, Merced, California 95343, USA; 3 Department of Developmental and Cell Biology, University of California, Irvine, California 92617, USA

## Abstract

State space truncation methods are widely used to approximate solutions of the chemical master equation. While most methods of this kind focus on truncating the state space directly, in this work, we propose modifying the underlying chemical reaction network by introducing *slack reactants* that indirectly truncate the state space. More specifically, slack reactants introduce an expanded chemical reaction network and impose a truncation scheme based on desired mass conservation laws. This network structure also allows us to prove inheritance of special properties of the original model, such as irreducibility and complex balancing. We use the network structure imposed by slack reactants to prove the convergence of the stationary distribution and first arrival times. We then provide examples comparing our method with the stationary finite state projection and finite buffer methods. Our slack reactant system appears to be more robust than some competing methods with respect to calculating first arrival times.

## I. INTRODUCTION

Chemical reaction networks (CRNs) are a fundamental tool in the modeling of biological systems, providing a concise representation of known chemical or biological dynamics. A CRN is defined by a family of chemical reactions of the form

$$\overset{n}{\underset{i=1}{\sum }}\alpha_{i}X_{i}\to \overset{n}{\underset{i=1}{\sum }}\beta_{i}X_{i},$$

where *α*_*i*_ and *β*_*i*_ are the number of species *X*_*i*_ consumed and produced in this reaction, respectively. The classical approach to modeling this CRN is to consider the concentrations $c\left(t\right)={\left(c_{1}\left(t\right),c_{2}\left(t\right),\ldots ,c_{n}\left(t\right)\right)}^{⊤}$, where *c*_*i*_(*t*) is the concentration of species *X*_*i*_ at time *t*, and to use a system of nonlinear differential equations to describe the evolution of the concentrations.

Suppose we are interested in studying the typical enzyme–substrate system^1^ given by the CRN

S+E⇌C→P+E, (1)

where the species *S*, *E*, *C*, and *P* stand for a substrate, an enzyme, an enzyme–substrate complex, and a product, respectively. The substrate and enzyme bind reversibly to form a substrate–enzyme complex, and the enzyme then acts upon the substrate forming the product. Given an initial condition where the molecular numbers of both *C* and *P* are 0, a natural question to ask is how long the system takes to produce the *first* copy of *P*. Similarly, there exists a time in the future where *S* and *C* are fully depleted, resulting in a chemically inactive system, and one can ask when this occurs. These are both quantities that the classical deterministic modeling leaves unanswered—by considering only continuous concentrations, there is no well-defined way to address modeling questions at the level of single molecules as the model assumes that all the reactions simultaneously occur within infinitesimally small time intervals.

Instead, by explicitly considering each individual copy of a molecule, we may formulate a continuous-time Markov chain. This stochastic modeling is especially important when the system consists of low copies of species, in which case the influence of intrinsic noise is magnified.^2–4^ Rather than deterministic concentrations *c*(*t*), we consider the continuous-time Markov chain $X\left(t\right)={\left(X_{1}\left(t\right),X_{2}\left(t\right),\ldots ,X_{d}\left(t\right)\right)}^{⊤}$ describing the molecular number of each species *X*_*i*_ at time *t*.

The questions regarding the enzyme–substrate system (1), such as the time of the first production of *P*, simply correspond to the *first passage times* of various combinations of states. For a continuous-time Markov process *X*, the first passage time to visit a set *K* of system states is formally defined as *τ* = inf{*t* ≥ 0 : *X*(*t*) ∈ *K*}.

One can directly compute *E*(*τ*) by using the transition rate matrix *A*. With few exceptions (such as CRNs with only zero- or first-order reactions^5^), most chemical reaction networks of any notable complexity will have an intractably large or infinite state-space, i.e., they exhibit the curse of dimensionality. This direct approach can, therefore, suffer from the high dimensionality of the transition rate matrix.

An alternative approach is to estimate the mean first passage time by generating sample trajectories with stochastic simulation algorithms such as the Gillespie algorithm.^6^ This overcomes the curse of dimensionality since a single trajectory needs only to keep track of its current population numbers. Nevertheless, there still remain circumstances under which it is more efficient to numerically evaluate the exact solution of the chemical master equation (CME)—in particular, when the Markov process rarely visits *K* so that significantly long simulations may be required to sample enough trajectories to estimate the mean first passage time.^7^

Fortunately, a broad class of state space reduction methods have recently been developed that allow for direct treatment of the transition rate matrix. These methods are based on the truncation-and-augmentation of the state space,^8–11^ the finite buffer method,^12,13^ and linear programming.^14,15^ A recent review summarizes truncation-based methods.^16^ The *stationary finite state projection (sFSP)*^11^ and the *finite buffer method*^12,13^ are examples of such truncation-based methods, which we will describe in detail below. They all satisfy provable error estimates on the probability distributions when compared with the original distribution. On the other hand, each of these methods has potential limitations for estimating mean first passage times and other quantitative features. For instance, using sFSP depends on the choice of a designated state, which can significantly alter the estimate for first passage times.

In this paper, we provide a new algorithm of state space reduction, the *slack reactant method*, for stochastically modeled CRNs. In this algorithm, we generate a new CRN from an existing CRN by adding one or multiple new species so that the associated stochastic system satisfies mass conservation relations and is confined to a finite number of states. For instance, we convert a simple birth and death model 0/ ⇌ *X* admitting an infinite state space to *Y* ⇌ *X* with the “slack reactant” *Y* to confine it on a finite state space. In order to ensure equivalent dynamics to the original system, we define a mild form of non-mass action kinetics for the new system. Since the state space reduction is implemented using a fully determined CRN, we can study the CRN using well-known tools of CRN theory such as deficiency zero and Lyapunov functions as long as they extend to this form of kinetics.

In Sec. III B, we provide an algorithm to produce a slack variable network, given a desired set of mass conservation relations. In addition to its theoretical uses, this algorithm allows us to implement existing software packages such as CERENA,^17^ StochDynTools,^18^ and FEEDME^19^ for chemical reaction networks to generate quantities such as the moment dynamics of the associated stochastic processes using the network structures as input.

We employ classical truncation Markov chain approaches to prove convergence theorems for slack networks. For fixed time *t*, if a probability density of each slack system under conservation quantity *N* converges to its stationary distribution uniformly in *N*, then the stationary distribution of the slack system converges to the original stationary distribution as *N* tends to infinity. We further prove that under a uniform tail condition of first passage times, the mean first passage time of the original system can be approximated with slack systems confined on a sufficiently large truncated state space. Finally, we show that the existence of the Lyapunov function for the original system guarantees that all the sufficient conditions for the first passage time convergence are satisfied. We also show that this truncation method is natural in the sense that a slack system admits the same stationary distribution up to a constant multiplication as the stationary distribution of the original system if the original system is complex balanced.

This paper is outlined as follows: In Sec. III, we introduce the slack reactant method and include several illustrative examples. In Sec. IV, we demonstrate that the slack method compares favorably with other state space truncation methods (sFSP and finite buffer method) when calculating mean first passage times. We prove convergence in the mean first passage time, and other properties, in Sec. V. In Sec. VII, we use slack reactants to estimate the mean first passage times for practical CRN models such as a Lotka–Volterra population model and a system of protein synthesis with a slow toggle switch.

## II. STOCHASTIC CHEMICAL REACTION NETWORKS

A chemical reaction network (CRN) is a graph that describes the evolution of a biochemical system governed by a number of *species* (S ) and *reactions* (R). Each node in the graph represents a possible state of the system, and nodes are connected by directed edges when a single reaction transforms one state into another. Each reaction consists of *complexes* and a *reaction rate constant*. For example, the reaction representing the transformation of complex *ν* to complex *ν*′ at rate *κ* is written as follows:

$$\nu \overset{\kappa }{\to }\nu^{\prime }.$$ (2)

A complex, such as *ν*, is defined as a number of each species $S_{i}\in S$. That is, *ν* = (*ν*_1_, *ν*_2_, …, *ν*_*d*_) representing a complex $\sum_{i=1}^{d}\nu_{i}S_{i}$, where *ν*_*i*_ ≥ 0 are the *stoichiometric coefficients* indicating how many copies of each species $S_{i}\in S$ belong in complex *ν*. The full CRN is thus defined by (S,C,R,Λ), where C and Λ represent the set of complexes and reaction propensities, respectively.

When intrinsic noise plays a significant role for system dynamics, we use a continuous-time Markov chain **X**(*t*) = (*X*_1_(*t*), *X*(2), …, *X*_*d*_(*t*)) to model the copy numbers of species *S*_*i*_ of a reaction network. The stochastic system treats individual reactions as discrete transitions between integer-valued states of the system. The probability density for *X*(*t*) is denoted as

$$p_{x_{0}}\left(x,t\right)=P\left(X\left(t\right)=x\;|\;X\left(0\right)=x_{0}\right),$$

where **X**(0) is the initial state. We occasionally denote by *p*(**x**, *t*) or *P*(*X*(*t*) = **x**) the probability omitting the initial state **x**_0_ when the contexts allow. For each state **x**, the probability density *p*(**x**) obeys the chemical master equation (CME), which gives the time-evolution of *p*(*t*) with a linear system of ordinary differential equations (ODEs),^20^

$$\frac{d}{dt}p^{⊤}\left(t\right)=p^{⊤}A.$$ (3)

Here, the entry *A*_*ij*_ (*i* ≠ *j*) is the transition rate at which the *i*th state transitions to the *j*th state. Letting **x** and **x**′ be the *i*th and *j*th states, the transition rate from **x** to **x**′ is

$$A_{ij}=\underset{\begin{array}{c} \nu \to \nu^{\prime }\\ x+\nu^{\prime }-\nu =x^{\prime } \end{array}}{\sum }\lambda_{\nu \to \nu^{\prime }}\left(x\right),$$

where *λ*_*ν*→*ν*′_ is the *reaction intensity* for a reaction *ν* → *ν*′.

The diagonal elements of *A* are defined as

$$A_{jj}=-\underset{i\neq j}{\sum }A_{ij}.$$

Regarding the reaction intensities, we will assume that

$$\lambda_{\nu \to \nu^{\prime }}\left(x\right)>0\text{ if and only if }x_{i}\geq \nu_{i}\text{ for each}i.$$ (4)

This is a slightly stronger condition than the so-called *stoichiometric compatibility*^21^ of the intensity function, namely, that *λ*_*ν*→*ν*′_(**x**) can only be positive if *x*_*i*_ ≥ *ν*_*i*_ for each *i*.

A typical choice for *λ*_*ν*→*ν*′_ is *mass*-*action*, which defines for **x** = (*x*_1_, *x*_2_, …, *x*_*d*_) and *ν* = (*α*_1_, *α*_2_, …, *α*_*d*_),

$$\lambda_{\nu \to \nu^{\prime }}\left(x\right)=\kappa_{\nu \to \nu^{\prime }}\overset{d}{\underset{k=1}{\prod }}x_{i}^{\left(\alpha_{i}\right)},$$

where *κ*_*ν*→*ν*′_ is the rate constant. Here, we used the notation $m^{\left(n\right)}=m\left(m-1\right)\left(m-2\right)\cdots \left(m-n+1\right)1_{\left\{n\geq m\right\}}$ for positive integers *m* and *n*.

A state **x** is called an *absorbing state* if *λ*_*ν*→*ν*′_ = 0 for all reactions *ν* → *ν*′. This means that if the Markov chain enters this state, it will remain there for all time. A Markov chain **X**(*t*) with **X**(0) = **x**_0_ is *accessible* to a state **x**_*T*_ if *P*(**X**(*t*) = **x**_*T*_ for some *t* | **X**(0) = **x**_0_) = 1.

## III. CONSTRUCTION OF SLACK NETWORKS

In this section, we introduce the method of slack reactants, which adds new species to an existing CRN so that the state space of the associated stochastic process is truncated to a finite subset of states. This model reduction accurately approximates the original system as the size of the truncated state space increases. We begin with a simple example to demonstrate the main idea of the method.

### A. Slack reactants for a simple birth-death model

Consider a mass-action birth-death model of a single species,
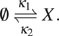
(5)For the associated stochastic process *X*, the mass-action assumption defines reaction intensities as *λ*_0/→*X*_(*x*) = *κ*_1_ and *λ*_*X*→0/_(*x*) = *κ*_2_*x* for each reaction in (5). Note that the count of species *X* could be any positive integer value as the birth reaction 0/ → *X* can occur unlimited times. Therefore, the state space for this system consists of infinitely many states. Consider instead the CRN
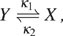
(6)where we have introduced the *slack reactant Y*. This new network admits *X* + *Y* = *N* as a *conservation law* for some *N* since for each reaction, either one species is degraded by one, while the other is produced by one.

Since the purpose of this new network is to approximate the qualitative behavior of the original system (5), we minimize the contribution of the slack reactant *Y* for modeling the associated stochastic system. Hence, we assign *Y* a special reaction intensity—instead of *λ*_*Y*→*X*_(*x*, *y*) = *κ*_1_*y* using mass-action, we choose

$$\lambda_{Y\to X}\left(x,y\right)=\kappa_{1}1_{\left\{y\geq 0\right\}},$$ (7)

and we use the same intensity *λ*_*X*→*Y*_(*x*, *y*) = *κ*_2_*x* for the reaction *X* → *Y*. By forcing *Y* to have “zero-order kinetics,” we ensure that the computed rates remain the same throughout the chemical network except for on the imposed boundary. This choice of reaction intensities not only preserves the conservation law **X**(*t*) + **Y**(*t*) = **X**(0) + **Y**(0) but also prevents the slack reactant *Y* from having negative counts with the characteristic term $1_{\left\{y\geq 0\right\}}$.

### B. Algorithm for constructing a network with slack reactants

In general, by using slack reactants, *any* conservation law can be deduced in a similar fashion to encode the desired truncation directly in the CRN. We have found this perspective to be advantageous with respect to studying complex CRNs. Rather than thinking about the software implementation of the truncation, it is often easier to design the truncation in terms of slack reactants and then implement the already-truncated system exactly.

We now provide an algorithm to automatically determine the slack reactants required for a specified truncation. It is often the case that a “natural” formulation arises (typically by replacing zero-order complexes with slack reactants), but when that is not the case, one can still systematically find a slack network by following our algorithm.

Consider any CRN (S,C,R,Λ) such that $S=\left\{X_{1},X_{2},\ldots ,X_{d}\right\}$ and $C=\left\{\nu^{j}\;|\;j=1,2,\ldots ,|C|\right\}$. Then, we define matrices associated with the given CRN.

1. Let *S* be the |C|×|R| *connectivity matrix* such that for each *r*,

$$-S_{ir}=S_{jr}=\left\{\begin{array}{c} 1\text{ if}r\text{th reaction is}\nu^{i}\to \nu^{j}\;\\ 0\text{ otherwise}.\; \end{array}\right.$$

2. Let *C* be the |S|×|C| *complex matrix* such that $C_{ij}=\nu_{i}^{j}$.

Suppose we wish to apply a number of *conservation bounds* to reduce the state-space of the associated chemical master equation, e.g., many equations of the form

$$w_{i,1}X_{1}+w_{i,2}X_{2}+\cdots +w_{i,d}X_{d}\leq N_{i},$$ (8)

for *i* = 1, 2, …, *m*. Then, we define additional matrices associated with the conservation bounds (8).

3. Let *W* be the *m* × *d* matrix of the conservation bounds in (8) such that *W*_*ij*_ = *w*_*i*,*j*_, and let $N={\left(N_{1},N_{2},\ldots ,N_{m}\right)}^{⊤}$.
4. For arbitrary positive integers *u*_*i*_, let *U* be an *m* × |*C*| matrix with each row of the form *u*_*i*_**1**^*⊤*^ = *u*_*i*_(1, 1, …, 1)^⊤^.
5. Define m×|C| matrix *D* = *U* − *WC*.

Finally, we use these matrices to define the slack network $\left(\tilde{S},\tilde{C},\tilde{R}\right)$.

6. Let $\tilde{S}=\left\{X_{1},\ldots ,X_{d},Y_{1},\ldots ,Y_{m}\right\}$.
7. Let $\tilde{C}=\left\{{\tilde{\nu }}^{j}\;|\;j=1,2,\ldots ,|C|\right\}$, where ${\tilde{\nu }}^{j}$ is the *j*th column of $\tilde{C}=\left(\begin{array}{c} C\\ D \end{array}\right)$.
8. Let $\tilde{R}=\left\{{\tilde{\nu }}^{i}\to {\tilde{\nu }}^{j}\;|\;-S_{ir}=S_{ij}=1\text{for some}r\right\}$.

We next verify that the newly generated network $\left(\tilde{S},\tilde{C},\tilde{R}\right)$ with the slack reactants *Y*_*i*_ admits conservation laws

$$w_{i,1}X_{1}+w_{i,2}X_{2}+\cdots +w_{i,d}X_{d}+Y_{i}=N_{i}$$ (9)

for each *i* = 1, 2, …, *m* so that the state space is truncated onto a finite set and the conservation bounds (8) hold. Note that for a given CRN with the matrices *S* and *C*, conservation laws **r**^*i*^ · *X* = *N*_*i*_ hold with conservation vectors **r**^1^, **r**^2^, …, **r**^*m*^ if and only if each reaction vector is orthogonal to **r**^*j*^. This is equivalent to *R*Γ = 0, where *R* is a matrix with rows ${r^{j}}^{⊤}$ and Γ = *CS* is the *stoichiometric matrix* whose columns indicate the reaction vector *ν*′ − *ν* associated with a reaction $\nu \to \nu^{\prime }\in R$.

Note that the network $\left(\tilde{S},\tilde{C},\tilde{R}\right)$ is generated with the connectivity matrix *S* and the new complex matrix $\tilde{C}$. Thus, the stoichiometric matrix of $\left(\tilde{S},\tilde{C},\tilde{R}\right)$ is $\tilde{\Gamma }=\tilde{C}S$. Then, since (1, 1, …, 1)*S* = **0**, we have

$$\left(\begin{array}{c} W\;I \end{array}\right)\tilde{\Gamma }=\left(\begin{array}{c} W\;I \end{array}\right)\tilde{C}S=\text{ 0}.$$

Therefore, (9) holds.

We model the stochastic system associated with the slack network by $X^{N}\left(t\right)=\left(X_{1}^{N}\left(t\right),X_{2}^{N}\left(t\right),\ldots ,X_{d}^{N}\left(t\right)\right)$, where each of the entries represents the count of species in the new network. Note that the count of each slack reactant *Y*_*i*_ is fully determined by species counts $X_{i}^{N}$’s because of the conservation law(s). As such, we do not explicitly model *Y*_*i*_’s.

The intensity function $\lambda_{\tilde{\nu }\to \tilde{\nu }}^{N}$ of *X*^**N**^ for a reaction $\tilde{\nu }\to {\tilde{\nu }}^{\prime }$ is defined as

$$\lambda_{\tilde{\nu }\to {\tilde{\nu }}^{\prime }}^{N}\left(x\right)=\lambda_{\nu \to \nu^{\prime }}\left(x\right)\overset{m}{\underset{i=1}{\prod }}1_{\left\{y_{i}\geq {\tilde{\nu }}_{d+1}\right\}},$$ (10)

where *y*_*i*_ = *N*_*i*_ − (*w*_*i*1_*x*_1_ + *w*_*i*1_*x*_1_ +⋯+ *w*_*id*_*x*_*d*_) and *ν* → *ν*′ is the reaction in R. Then, we denote by $\left(\tilde{S},\tilde{C},\tilde{R},\Lambda^{N}\right)$ a new system with slack reactants obtained from the algorithm, where $K_{N}$ is the collection of kinetic rates $\left\{\lambda_{\tilde{\nu }\to {\tilde{\nu }}^{\prime }}^{N}:\tilde{\nu }\to {\tilde{\nu }}^{\prime }\in \tilde{R}\right\}$. We refer this system with slack reactants to a *slack system*.

Here, we highlight that the connectivity matrix *S* and the complex matrix *C* of the original network are preserved for a new network with slack reactants. Thus, the original network and the new network obtained from the algorithm have the same connectivity. This identical connectivity allows the qualitative behavior of the original network, which solely depends on *S* and *C*, to be inherited to the new network with slack reactants. We particularly exploit the inheritance of accessibility and the inheritance of Poissonian stationary distribution in Sec. VI.

Remark III.1.
*A single conservation relation, such as* **w** · *X* + *y* = *N with a non-negative vector* **w***, is sufficient to truncate the given state space into a finite state space. Hence, in this manuscript, we mainly consider a slack network that is obtained with a single conservation vector* **w** *and a single slack reactant Y*.

Remark III.2.
*Although we primarily think about bounding our species counts from above, we could also bound species counts from below by choosing negative integers for w*_*i*,*j*_*. For instance, suppose that the stochastic process X associated with the birth-death model* (5) *satisfies X*(0) = 100*. If we are interested in computing the first hitting time to state 50, then by adding a slack reactant Y*, *we can truncate the state space of X from below with a conservation law* −*X*(*t*) + *Y*(*t*) = −*X*(0) + *Y*(0) = −40 *so that X*(*t*) ≥ 40 *for each t. In this case, a slack network is X* + *Y* ⇌ 0 *with Y*(0) = 60.

Example III.1.
We illustrate our slack algorithm with an example CRN consisting of two species and five reactions indicated by edge labels on the following network:
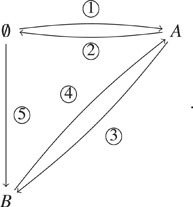
(11)We enumerate the complexes in the order of 0/, *A*, and *B*. We order the reactions according to their labels on the network. Thus, the connectivity matrix *S* and complex matrix *C* are defined as follows:

$$S=\left[\begin{array}{ccccc} -1 & 1 & 0 & 0 & -1\\ 1 & -1 & -1 & 1 & 0\\ 0 & 0 & 1 & -1 & 1 \end{array}\right],$$

$$C=\left[\begin{array}{ccc} 0 & 1 & 0\\ 0 & 0 & 1 \end{array}\right].$$

Suppose we set a conservation bound *A* + *B* ≤ *N* for some *N* > 0. Then, the matrix $W=\left[\begin{array}{cc} 1 & 1 \end{array}\right]$and $D=\left[\begin{array}{ccc} u & u & u \end{array}\right]-\left[\begin{array}{ccc} 0 & 1 & 1 \end{array}\right]$for an arbitrary positive integer *u*.

When *u* = 1, the network with the slack reactant *Y* is
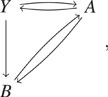
(12)where we have the conservation relation *A* + *B* + *Y* = *N*, where *N* = *A*(0) + *B*(0) + *Y*(0).

When *u* = 2, the network with the slack reactant *Y* is
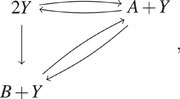
(13)where we have the same conservation relation *A* + *B* + *Y* = *N*.

Here, we explain why network (12) is preferred to (13) because it is less intrusive. Let **X** = (*X*_*A*_, *X*_*B*_, *X*_*Y*_) and **X**′ = ($X_{A}^{\prime },X_{B}^{\prime },X_{Y}^{\prime }$) be the stochastic process associated with networks (12) and (13), respectively. Suppose that the initial state of both **X** and **X**′ is (0, 0, *N*). **X** can reach the state (0, *N*, 0) by transitioning *N* times with the reaction *Y* → *B*. This state corresponds to the state (0, *N*) in the original network (11). On the other hand, **X**′ cannot reach the state (0, *N*, 0). This is because the states (0, 0, *N*) and (0, *N* − 1, 1) are the only states from which **X**′ jumps to (0, 0, *N*). However, no reaction in (13) can be fired at the states since no species *Y* presents at those states.

Consequently, one state, which is accessible in the original network (11), is lost in the system associated with network (13). However, it can be shown that the stochastic process associated with network (12) preserves all the states of the original network. This occurs mainly because the matrix *D* for network (12) is sparser than the matrix *D* for network (13). We discuss how to minimize the effect of slack reactants in Sec. III C.

### C. Optimal slack CRNs for effective approximation of the original systems

The algorithm we introduced to construct a network with slack reactants is valid and unique up to any user-defined conservation bounds (8), and the outcome is the matrix *D* that indicates the stoichiometric coefficient of slack reactants at each complex.

As we showed in Example III.1, to minimize the “intrusiveness” of a slack network, we can simplify a slack network by setting as many *D*_*ij*_ = 0 as possible. To do that, we choose the entries of **u** such that *u*_*i*_ is the maximum entry of the *i*th row of *AC*. We further optimize the effect of the slack reactants by removing the “redundant” stoichiometric coefficient of slack reactants. For example, for a CRN,

0/⇌A→2A, (14)

the algorithm generates the following new CRN with a single slack reactant *Y*:

2Y⇌A+Y→2A. (15)

However, by breaking up the connectivity, we can also generate another network

Y⇌A, A+Y→2A. (16)

The network in (15) is more intrusive than the network in (16) in the sense of accessibility. At any state where *Y* = 0, the system associated with (15) will remain unchanged because no reaction can take place. However, the reaction *A* → *Y* in (16) can always occur despite *Y* = 0. Hence, (16) preserves the accessibility of the original system associated with (14) as any state for *A* is accessible from any other state in the original reaction system (14). We refer such a system with slack reactants generated by canceling redundant slack reactants to an *optimized slack system*. In Sec. VI, we explore the accessibility of an optimized reaction network with slack reactants in a more general setting.

Finally, we can make a network with slack reactants admit a better approximation of a given CRN by choosing an optimized conservation relation in (8). First, we assume that only a single conservation law and a single slack reactant are added to a given CRN. For the purpose of state space truncation onto finitely many states, a single conservation law is enough as all species could be bounded by *N*, as shown in (8). Let this single conservation law be

$$w_{1}X_{1}+w_{2}X_{2}+\cdots w_{d}X_{d}+Y=N.$$

**FIG. 1.**
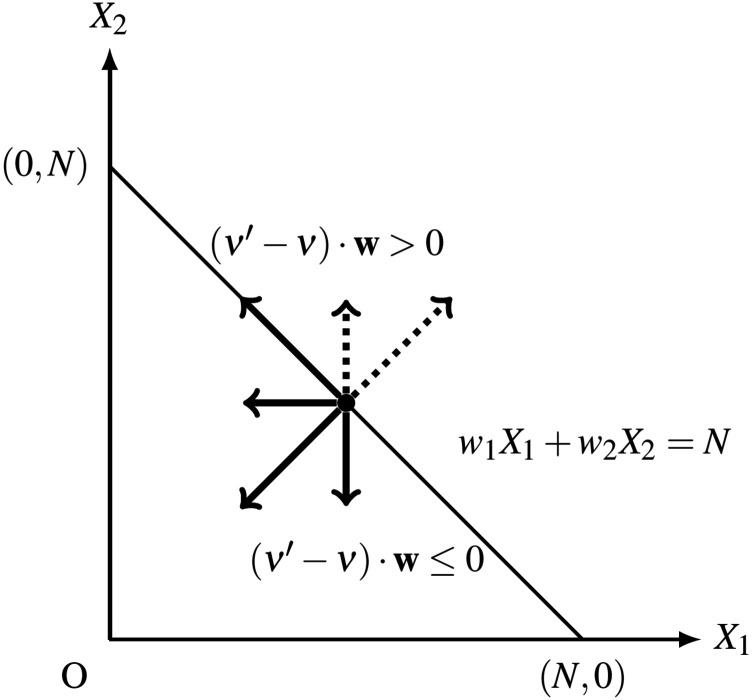
The dotted arrows correspond to the reaction vectors that are turned off (i.e., the associated reaction intensity is zero) at the boundary of the state space for a slack system, as described in Sec. III C.

Then, the matrix *W* defined in Sec. III B is a vector ${\left(w_{1},w_{2},\ldots ,w_{d}\right)}^{⊤}$, and we denote this by **w**. By the definition of the intensities (10) for a network with slack reactants, some reactions are turned off when *Y* = 0, i.e., *w*_1_*X*_1_ +⋯+ *w*_*d*_*X*_*d*_ = *N*. Geometrically, a reaction outgoing from the hyperplane *w*_1_*X*_1_ +⋯+ *w*_*d*_*X*_*d*_ = *N* is turned off (Fig. 1).

Hence, we optimize the estimation with slack reactants by minimizing such intrusiveness of turned off reactions. To do that, we choose **v**, which minimizes the number of the reactions in $\left\{\nu \to \nu^{\prime }\in R:\left(\nu^{\prime }-\nu \right)\cdot w>0\right\}$.

## IV. COMPARISON TO OTHER TRUNCATION-BASED METHODS

In this section, we demonstrate that our method can potentially resolve limitations in calculating mean first passage times observed in other methods of state space truncation, namely, sFSP and the finite-buffer method. Both methods require the user to make decisions about the state-space truncation that may introduce variability in the results. While all methods will converge to the true result as the size of the state space increases, we show that our method is less dependent on user-defined quantities. This minimizes additional decision-making on the part of the user that can lead to suboptimal results, especially in a context where the solution of the original system is not known.

### A. Comparison to the sFSP method

**FIG. 2.**
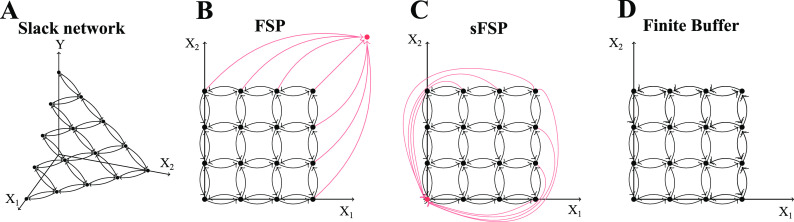
Schematic images of various state-space truncation methods for a CRN with two species: (a) slack network (this paper), (b) Finite State Projection (FSP),^10^ (c) stationary Finite State Projection (sFSP),^11^ and (d) finite buffer method.^12,13^

A well-known state truncation algorithm is known as the *Finite State Projection* (FSP) method.^10^ For a given continuous-time Markov chain, the associated FSP model is restricted to a finite state space. If the process escapes this truncated space, the state is sent to a designated absorbing state [see Fig. 2(b)]. For a fixed time *t*, the probability density function of the original system can be approximated by using the associated FSP model with sufficiently many states. The long-term dynamics of the original system, however, is not well approximated because the probability flow of the FSP model leaks to the designated absorbing state in the long run.

**FIG. 3.**
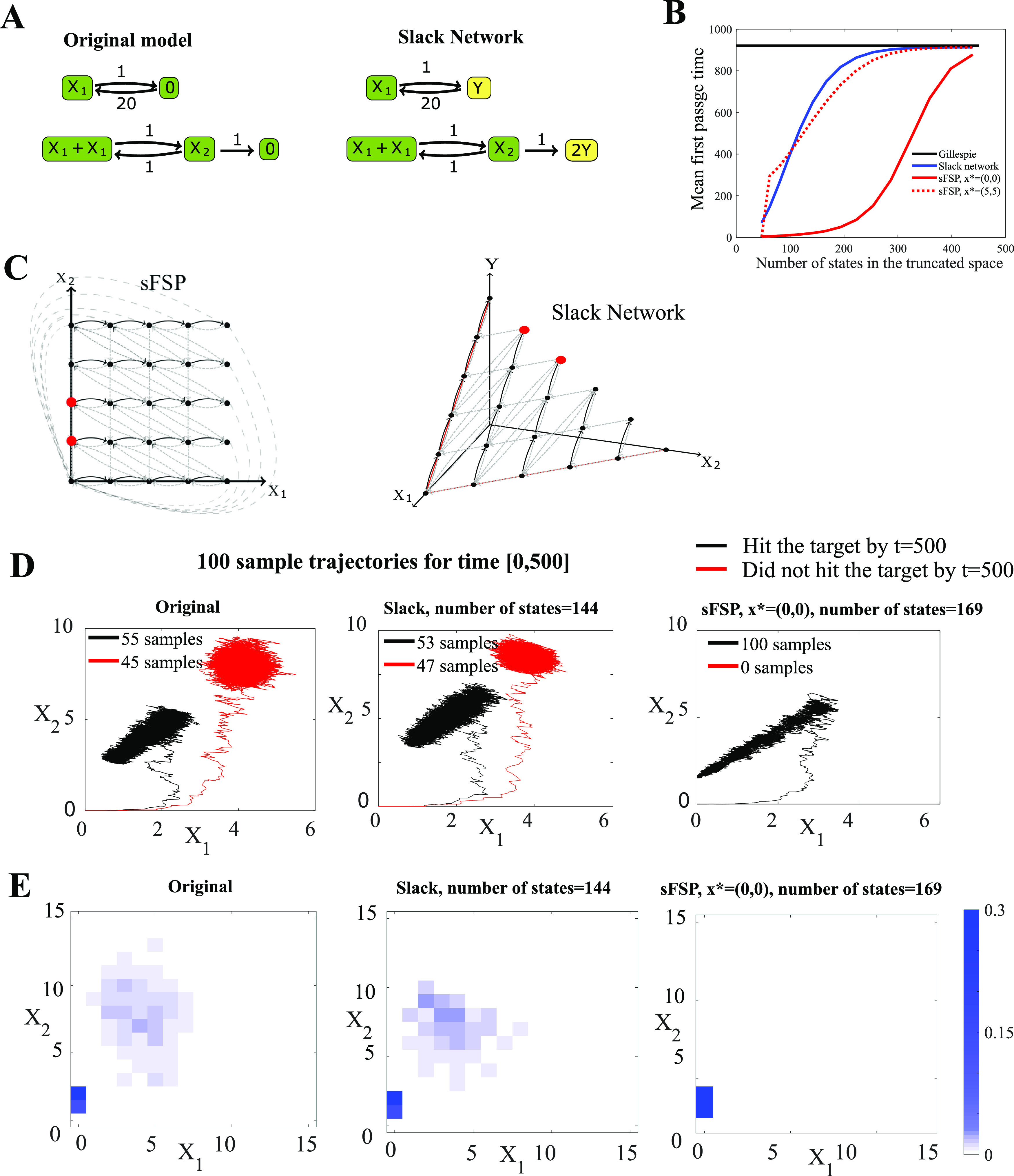
Comparison between an sFSP model and a slack network for the same reaction system. (a) Original 2-dimensional system and its slack network. (b) Mean first passage time as a function of the truncated state size, comparing sFSP and the slack reactant method. (c) The state space truncation corresponding to the sFSP method and the slack method for this model. Target states for the first passage time are indicated in red. (d) Mean of 100 sample trajectories obtained by Gillespie simulations for the original system, the slack system, and the sFSP system. The red lines indicate the mean of the trajectories that have touched the target states within [0, 500], while the black lines are the mean of the trajectories that did not touch the target space during this time. (e) Probability density heat maps of the stochastic processes modeled under the original system, the slack system, and the sFSP model, respectively, at *t* = 500 (see Sec. IV A for more information).

To fix this limitation of FSP, Gupta *et al.* proposed the *stationary Finite State Projection* (sFSP) method.^11^ This method also projects the original state space onto a finite state space as the FSP method intended to. However, sFSP does not create a designated absorbing state as all outgoing transitions from the projected finite state space are merged to a single state x^*^ “inside” the finite state space [Fig. 2(c)]. The sFSP has been frequently used to estimate the long-term distribution of discrete stochastic models. However, if the size of the truncated state space is not sufficiently large, this method could fail to provide accurate estimation for the first passage time. To demonstrate this case, we consider the following simple 2-dimensional model. In the network shown in Fig. 3(a), two *X*_1_ proteins are dimerized into protein *X*_2_, while *X*_1_ is being produced at a relatively high rate. The state space of the original model is the full 2-dimensional positive integer grid. We estimate the time until the system reaches one of the two states indicated in red in Fig. 3(c), and we use alternative methods to do this.

For the sFSP, we project the original state space onto the rectangle by restricting *X*_1_ ≤ *N* and *X*_2_ ≤ *N* for some *N* > 0, and we fix the origin (0, 0) as the designated state x^*^ [Fig. 3(c)]. If the process associated with the sFSP model escapes the rectangle, it transports to the designated state immediately. On the other hand, we also consider a slack network shown in Fig. 3(b), where we introduce the conservation law *X*_1_ + 2*X*_2_ + *Y* = *N* for some *N* > 0. Let *τ* = inf{*t* ≥ 0:*X*_1_ = 1 and *X*_2_ ∈ {1, 2}} be the first passage time we want to estimate.

For a Markov chain defined on a finite state space, the mean first passage time is computable with the inverse of an absorbing transition matrix, as detailed in Ref. 22 (see Appendix A for details). Hence, by using the inverse absorbing matrix for each truncation method, we obtain the mean of *τ* by using different values of *N*. We also obtain an “almost” true mean of *τ* by using 10^5^ Gillespie simulations of the original process. As shown in Fig. 3(b), the slack network model provides a more accurate mean first passage time estimation for the size of truncation in between 100 and 400 if the designated return state of sFSP is (0, 0).

The inaccurate estimate from the sFSP is due to the choice of a return state. The sFSP model escapes the confined space often because the production rate of *X*_1_ is relatively high. When it returns back to the origin, it is more likely to visit the target states {*X*_1_ = 1 and *X*_2_ ∈ {1, 2}} than the original process.

Figure 3 shows that the mean first passage time of this system using sFSP depends significantly on the location of the chosen designated state. One of the two states is a particularly poor choice for sFSP, but it illustrates the idea that without previous knowledge of the system, it can be difficult to know which states will perform well.

We display the behavior of individual solutions of the original model, the slack network, and the sFSP model in Fig. 3(d). The trajectory plots show that within the time interval [0, 500], almost half of the 100 samples from both the original model and the slack network model stay far away from the target states, while all 100 sample trajectories from the sFSP model stay close to the target states. We also illustrate this point in Fig. 3(e) with heat maps of the three models at *t* = 500. Note that only in the case of the sFSP, the probability densities are concentrated at the target states.

### B. Comparison to the finite buffer method

The finite buffer method was proposed to estimate the stationary probability landscape with state space truncation and a novel state space enumeration algorithm.^12,13^ For a given stochastic model associated with a CRN, the finite buffer method sets inequalities among species such as (8), so-called buffer capacities. Then, at each state **x**, the transition rate of a reaction *ν* → *ν*′ is set to be zero if at least one of the inequalities does not hold at **x** + *ν*′ − *ν*. We note that the algorithm, described in Sec. III B, for generating a slack network uses the same inequalities. Thus, the finite buffer method and the slack reactant method truncate the state space in the same way. We have shown, in Sec. III C, that this type of truncation can create “additional” absorbing states. These additional absorbing states change the accessibility between the states, which means the mean first passage times cannot be accurately estimated. However, the regular slack systems preserve the network structure of the original network. Hence, we are able to prove, as we already noted, that regular slack networks inherit the accessibility of the original network as long as the original network is “weakly reversible,” as we will define below.

We demonstrate this disparity between the finite buffer and slack methods with the following network. Consider the mass-action system (11) with a fixed initial state **x**_0_ = (*a*_0_, *b*_0_). We are interested in estimating the mean first passage time to a target state **x**_*T*_ = (10, 10). Note that the state space is irreducible (i.e., every state is accessible from any other state) as the network consists of unions of cycles. This condition, the union of cycles, is precisely what is meant by weakly reversible.^23,24^ Thus, the original stochastic system has no absorbing state and is accessible to the target state **x**_*T*_. Therefore, it is critical for a state space truncation method not to create an additional absorbing state so that the reduced system still can reach the target state with probability 1.

To use the finite buffer method on this network, we set 2*X*_*A*_ + *X*_*B*_ ≤ *N* as the buffer capacity, where **X** = (*X*_*A*_, *X*_*B*_) is the associated stochastic process. (Here, we choose *N* > 30, so the state space contains the target state **x**_*T*_.) Hence, when *X* satisfies 2*X*_*A*_ + *X*_*B*_ = *N*, the reactions 0/ → *A*, *B* → *A*, and 0/ → *B* cannot be fired as 2*X*_*A*_ + *X*_*B*_ exceeds the buffer capacity. We now demonstrate that the system has a new absorbing state. By first using reaction *A* → *B*, to deplete all *A*, and then 0/ → *B*, every state can reach the state (0, *N*) in finite time with positive probability. The state (0, *N*) is the absorbing state because no other reactions can occur. Reactions *A* → 0/ and *A* → *B* require at least one *A* species, and any other reactions lead to states exceeding the buffer capacity. Therefore, the finite buffer method has introduced a new absorbing state not present in the original model so that the system can be trapped at (0, *N*) with positive probability, and in turn, it is not accessible to **x**_*T*_. Therefore, the mean first passage time to **x**_*T*_ is infinite under the finite buffer method.

Now, we show that the explicit network structure of our slack network formulation will preserve the accessibility of the original system. We consider the same inequality 2*X*_*A*_ + *X*_*B*_ ≤ *N* as above with *N* > 30. We generate the slack network by using the algorithm shown in Sec. III B,
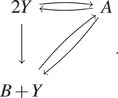
(17)Note that the associated stochastic process **X** = (*X*_*A*_, *X*_*B*_, *Y*) admits the conservation relation 2*X*_*A*_ + *X*_*B*_ + *Y* = *N*, implying that 2*X*_*A*_ + *X*_*B*_ ≤ *N*. The state (0, *N*, 0) cannot be reached as the only state that is accessible to (0, *N*, 0) is (0, *N* − 1, 2), but it violates the conservation law.

As we highlighted in Sec. III C, slack networks preserve the connectivity of the original network (11); hence, network (17) is also weakly reversible. Thus, the state space of the stochastic process associated with the slack network is irreducible by Corollary VI.1. This implies that there is no absorbing state and the system is accessible to **x**_*T*_, unlike the stochastic process associated with the finite buffer relation. See Sec. VI for more details about the accessibility of slack systems.

## V. CONVERGENCE THEOREMS FOR SLACK NETWORKS

In this section, we establish theoretical results on the convergence of properties of a slack network to the original network. (Proofs of the theorems below are provided in Appendix B.) Many of these results rely on theorems from Hart and Tweedie^25^ who studied when the probability density function of a truncated Markov process converges to that of the original Markov process. We employ the same idea of their proof to show the convergence of a slack network to the original network.

By assuming “uniform mixing,” we show the convergence of the stationary distribution of the slack system to the stationary distribution of the original system as the conservation quantity *N* grows. Furthermore, we show the convergence of mean first passage times for the slack network to the true mean first passage times. In particular, all these conditions hold when there is a Lyapunov function for the original system.

In this section, assume that a given CRN (S,C,R,Λ) is well defined for all time *t* and let $\left(\tilde{S},\tilde{C},\tilde{R},\Lambda^{N}\right)$ be an associated slack network obtained with a single conservation quantity **w** · **X** ≤ *N*. We denote by *X* and **X**^*N*^ the associated stochastic processes of the original CRN and the slack network, respectively. We fix the initial state for both systems, i.e., **X**(0) = **X**^*N*^(0) = **x**_0_ for some **x**_0_ and for each *N*. [This means that we can only consider slack systems where *N* is large enough so that **w** · **x**(0) < *N*.] Assume that both the original and slack systems are irreducible, and we denote by S and $S_{N}$ the state spaces for each, respectively. (In Sec. VI, we prove accessibility properties that the slack system can inherit from the original system.)

Note that every state in $S_{N}$ satisfies our conservation inequality. That is, for every $x\in S_{N}$, we have **w** · **x** ≤ *N*. It is possible that $S_{N}=S_{N+1}$ for some *N*. For simplicity, we assume that the truncated state space is always enlarged with respect to the conservation quantity *N*, that is, $S_{N}\subset S_{N+1}$ for each *N*. (For the general case, we could simply consider a subsequence *N*_*k*_ such that *N*_*k*_ < *N*_*k*+1_ and $S_{N_{k}}\subset S_{N_{k+1}}$ for each *k*.)

As defined in Sec. II, *λ*_*ν*→*ν*′_ ∈ Λ is the intensity of a reaction *ν* → *ν*′ for the associated stochastic system *X*. We also denote by $\lambda_{\tilde{\nu }\to {\tilde{\nu }}^{\prime }}^{N}\in \Lambda^{N}$ the intensity of a reaction $\tilde{\nu }\to {\tilde{\nu }}^{\prime }$ for the associated stochastic system **X**^*N*^. Finally, we let *p*(**x**, *t*) and *p*_*N*_(**x**, *t*) be the probability density function of *X* and **X**^*N*^, respectively. We begin with the convergence of the probability density functions of the slack network to the original network with increasing *N*.

Theorem V.1. *For any* $x\in S_{N}$ *and T* ≥ 0*, we have*

$$\underset{N\to \infty }{\lim}\underset{t\in \left[0,T\right]}{\sup}|p\left(x,t\right)-p_{N}\left(x,t\right)|=0.$$

A Markov process defined on a finite state space admits a stationary distribution. Hence, **X**^*N*^ admits a stationary distribution *π*_*N*_. If the slack system satisfies the condition of “uniform mixing, that is, the convergence rate of ‖*p*_*N*_(**x**, *t*) − *π*_*N*_(**x**)‖_1_ is uniform in *N*, then we have the following result.

Theorem V.2. *Suppose that* **X** *admits a stationary distribution π. Suppose further that there exists a positive function h*(*t*)*, which is independent of N, such that* ‖*p*_*N*_(·, *t*) − *π*_*N*_‖_1_ ≤ *h*(*t*) *and* $\underset{t\to \infty }{\lim}h\left(t\right)=0$*. Then,*

$$\underset{N\to \infty }{\lim}\|\pi -\pi_{N}{\|}_{1}=0.$$

We now consider the convergence of the mean first passage time of **X**^*N*^. Recall that we assumed that both stochastic processes have the same initial state **X**(0) = **X**^*N*^(0) = **x**_0_ and both state spaces S and $S_{N}$ are irreducible. Hence, for any K⊆S, each state in $S_{N}$ is accessible to *K* for sufficiently large *N*.

Theorem V.3. *For a subset K of the state space of* **X***, let τ and τ*_*N*_ *be the first passage times to K for* **X** *and to* $K\cap S_{N}$ *for* **X**^*N*^*, respectively. Assume the following conditions:*

1. **X** *admits a stationary distribution π.*
2. $\underset{N\to \infty }{\lim}\|\pi -\pi_{N}{\|}_{1}=0$*.*

*Then, for any T* ≥ 0*,*

$$\underset{N\to \infty }{\lim}\underset{t\in \left[0,T\right]}{\sup}|P\left(\tau >t\right)-P\left(\tau_{N}>t\right)|=0.$$

*If we further assume that*

3. *E*(*τ*) < ∞.
4. *There exists g*(*t*) *such that P*(*τ*_*N*_ > *t*) ≤ *g*(*t*) *for all N and* $\int_{0}^{\infty }g\left(t\right)dt<\infty$*.*

*Then,*

$$\underset{N\to \infty }{\lim}|E\left(\tau \right)-E\left(\tau_{N}\right)|=0.$$

Remark V.1.
*To obtain the convergence of higher moments of the first passage time, we need only replace conditions E*(*τ*_*K*_) < ∞ *and* $\int_{0}^{\infty }g\left(t\right)dt<\infty$ *with*

$$E\left(\tau_{K}^{n}\right)<\infty \text{ and }\int_{0}^{\infty }g\left(t^{\frac{1}{n}}\right)dt<\infty ,$$ (18)

*respectively.*

We now show that if a Lyapunov function exists for the original system, the conditions in Theorem V.2 and Theorem V.3 hold. The Lyapunov function approach was proposed by Meyn and Tweedie,^26^ and it has been used to study long-term dynamics of Markov processes,^11,27–29^ especially exponential ergodicity. Gupta *et al.*^11^ used a linear Lyapunov function to show that the stationary distribution of an sFSP model converges to a stationary distribution of the original stochastic model and used the Lyapunov function to explicitly compute the convergence rate. In particular, we show Lyapunov functions exist for the examples we consider in Sec. VII.

Theorem V.4. *Suppose that there exists a function V and positive constants C and D such that for all* **x***,*

1. *V*(**x**) ≥ 1 *for all* x∈S*,*
2. *V is an increasing function in the sense that*

$$V\left(x_{N+1}\right)\geq V\left(x_{N}\right)$$

*for each* $x_{N+1}\in S_{N+1}\backslash S_{N}$ *and* $x_{N}\in S_{N}$, *and*

3. $$\underset{\nu \to \nu^{\prime }\in R}{\sum }\lambda_{\nu \to \nu^{\prime }}\left(x\right)\left(V\left(x+\nu^{\prime }-\nu \right)-V\left(x\right)\right)\leq -CV\left(x\right)+D.$$

*Then, the conditions in Theorem V.3 hold.*

Remark V.2.
*Conditions* (18) *hold if a Lyapunov function satisfying the conditions in Theorem V.4 exists. Thus, the convergence of the higher moments of the first passage time also follows.*

## VI. INHERITANCE OF SLACK NETWORKS

As we showed in Sec. IV B, not all state space truncations preserve accessibility of states in the original system. (For the example in Sec. IV B, the truncation created a new absorbing state.) Thus, it is desirable to obtain reduced models that are guaranteed to maintain the accessibility of the original system to predetermined target states. In this section, we show that under mild conditions, both a regular slack system and an optimized slack system preserve the original accessibility. The proofs of the theorems introduced in this section are in Appendix C. The key to these results is the condition of weak reversibility.

Definition VI.1.
*A reaction network is* ***weakly reversible*** *if each connected component of the network is strongly connected. That is, if there is a path of reactions from a complex ν to ν*′*, then there is a path of reactions ν*′ *to ν*.

We note that the weakly reversible condition applies to the network graph of the CRN. The network graph consists of complexes (nodes) and reactions (edges). It is a sufficient condition for irreducibility of the associated mass-action stochastic process. Indeed, the sufficiency of weak reversibility holds even under general kinetics as long as condition (4) is satisfied.^30^ Hence, irreducibility of a regular slack network follows since it preserves weak reversibility of the original network, and the kinetics modeling the regular slack system satisfies (4).

Corollary VI.1.
*Let* (S,C,R,Λ) *be a weakly reversible CRN with intensity functions* Λ = {*λ*_*ν*→*ν*′_} *satisfying* (4)*. Then, the state space of the associated stochastic process with a regular slack network* $\left(\tilde{S},\tilde{C},\tilde{R},\Lambda^{N}\right)$ *is a union of closed communication classes for any N*.

In case the original network is not weakly reversible, we can still guarantee that optimized slack systems have the same accessibility as the original system, provided that all species have a degradation reaction (*S*_*i*_ → 0/).

Theorem VI.1. *Let* (S,C,R,Λ) *be a reaction network such that* $\left\{S_{i}\to 0\text{/}:S_{i}\in S\right\}\subset R$*. Suppose that the stochastic process X associated with* (S,C,R,Λ) *and beginning at the point* **x**_0_ *is irreducible. Let* **X**^*N*^ *be the stochastic process associated with an optimized slack network* $\left(\tilde{S},\tilde{C},\tilde{R},\Lambda^{N}\right)$ *such that* **X**^*N*^(0) = **x**_0_ *for every N large enough. Then, for any subset K of the state space of* **X***, there exists N*_0_ *such that* **X**^*N*^ *reaches K almost surely for N* ≥ *N*_0_.

This theorem follows from the fact that a slack system only differs from the original system when it runs out of slack reactants. However, in an optimized slack system, degradation reactions are allowable with no slack reactants. Hence, our proof of Theorem VI.1 relies on the presence of all degradation reactions.

A slack network may also inherit its stationary distribution from the original reaction system. When the original system admits a stationary distribution of a product form of Poisson distributions under the *complex balance* condition, a slack system inherits the same form of the stationary distribution as well. A reaction system is complex balanced if the associated deterministic mass-action system admits a steady state c^*^ such that

$$\underset{\begin{array}{c} \nu \in C\\ \nu \to \nu^{\prime }\in R \end{array}}{\sum }f_{\nu }\left(c^{*}\right)=\underset{\begin{array}{c} \nu^{\prime }\in C\\ \nu \to \nu^{\prime }\in R \end{array}}{\sum }f_{\nu^{\prime }}\left(c^{*}\right),$$

where $f_{\nu }\left(x\right)=\kappa_{\nu \to \nu^{\prime }}x_{1}^{\nu_{1}}\cdots x_{d}^{\nu_{d}}$ is the deterministic mass-action rate^31^ with a rate constant *κ*_*ν*→*ν*′_. If a reaction system is complex balanced, then its associated stochastic mass-action system admits a stationary distribution corresponding to a product of Poisson distributions centered at the complex balance steady state.^32^ The following lemma shows that the complex balancing of the original network is inherited by a regular slack network.

Lemma VI.1.
*Suppose that* (S,C,R,Λ) is a reaction network whose mass-action deterministic model admits a complex balanced steady state c^*^. Then, any regular slack network $\left(\tilde{S},\tilde{C},\tilde{R},\Lambda^{N}\right)$ *with slack reactants Y*_1_, …, *Y*_*m*_ *also admits a complex balanced steady state at* $\tilde{c}={\left(c^{*},1,1,\ldots ,1\right)}^{⊤}$.

Remark VI.1.
*Note that a regular slack network also preserves the deficiency of the original network. Deficiency δ of a reaction network is an index such that*

δ=n−ℓ−s,

*where n is the number of the complexes, ℓ is the number of connected components, and s is the rank of the stoichiometric matrix of the reaction network. Deficiency characterizes the connectivity of the network structure, and surprisingly, it can also determine the long-term behavior of the system dynamics regardless of the system parameters.*^31–33^ *A regular slack network and original network have the same number of complexes n and the same connectivity matrix S, which implies they have the same number of connected components ℓ. Furthermore, using the notation from* Sec. III B*, the stoichiometric matrices are* Γ = *CS for the original network and*

$$\tilde{\Gamma }=\left(\begin{array}{c} C\\ U-AC \end{array}\right)S=\left(\begin{array}{c} CS\\ -WCS \end{array}\right)$$

*for a slack network, which means that they have the same rank s. Together, these imply that the original network and its regular slack network have the same deficiency.*

Since the complex balancing is inherited with the same steady state values for *X*_*i*_, we have the following stochastic analog of inheritance of the Poissonian stationary distribution for regular slack systems.

Theorem VI.2. *Let* **X** *be the stochastic mass-action system associated with a complex balanced* (S,C,R,Λ) *with an initial condition* **X**(0) = **x**_0_*. Let* **X**^*N*^ *be the stochastic system associated with a regular slack system* $\left(\tilde{S},\tilde{C},\tilde{R},\Lambda^{N}\right)$ *with* **X**^*N*^(0) = **x**_0_. *Then, for the state space* $S_{N}$ *of* **X**^*N*^*, there exists a constant M*_*N*_ > 0 *such that*

$$\pi_{N}\left(x\right)=M_{N}\pi \left(x\right)\text{ for}x\in S_{N},$$

*where π and π*_*N*_ *are the stationary distributions of* **X** *and* **X**^*N*^*, respectively.*

We demonstrate Lemma VI.1 and Theorem VI.2 with a simple example.

Example VI.1.
Consider two networks,
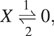
(19)
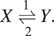
(20)Let *X* and *X*^*N*^ be systems (19) and (20), respectively, where *N* is the conservation quantity *X*^*N*^(*t*) ≤ *N*. Under mass-action kinetics, the complex balance steady state of (19) is c^*^ = 2. Under mass-action kinetics, $\tilde{c}=\left(2,1\right)$ is a complex balance steady state of (20).

Now, let *π* and *π*_*N*_ be the stationary distribution of *X* and *X*^*N*^, respectively. By Theorem 6.4, Ref*.* 32, *π* is a product form of Poisson distributions such that

$$\pi \left(x\right)=e^{-2}\frac{{c^{*}}^{x}}{x!}\text{ for each state}x.$$

By plugging *π* into the chemical master equation (3) of *X*^*N*^ and showing that for each **x**

$$\begin{array}{ll} & \lambda_{X\to Y}^{N}\left(x+1\right)\pi \left(x+1\right)+\lambda_{Y\to X}^{N}\left(x-1\right)\pi \left(x-1\right)\\ & \;=\left(x+1\right)1_{\left\{N-x-1\geq 0\right\}}e^{-2}\frac{2^{x+1}}{\left(x+1\right)!}+21_{\left\{N-x+1\geq 1\right\}}e^{-2}\frac{2^{x-1}}{\left(x-1\right)!}\\ & \;=x1_{\left\{N-x\geq 0\right\}}e^{-2}\frac{2^{x}}{x!}+21_{\left\{N-x\geq 1\right\}}e^{-2}\frac{2^{x}}{x!}\\ & \;=\lambda_{X\to Y}^{N}\left(x\right)\pi \left(x\right)+\lambda_{Y\to X}^{N}\left(x\right)\pi \left(x\right), \end{array}$$

we can verify that *π* is a stationary solution of the chemical master equation (3) of *X*^*N*^. Since the state space of *X*^*N*^ is $\left\{x\in Z_{\geq 0}:x\leq N\right\}$, we choose a constant *M*_*N*_ such that

$$\underset{0\leq x\leq N}{\sum }M_{N}\pi \left(x\right)=1.$$

Then, *π*_*N*_ = *M*_*N*_*π* is the stationary distribution of *X*^*N*^.

## VII. APPLICATIONS OF SLACK NETWORKS

In this section, we demonstrate the utility of slack reactants in computing mean first-passage times for two biological examples. For both examples, we compute the mean first passage time via the matrix inversion approach, as shown in Appendix A.

### A Lotka–Volterra model with migration

A.

**FIG. 4.**
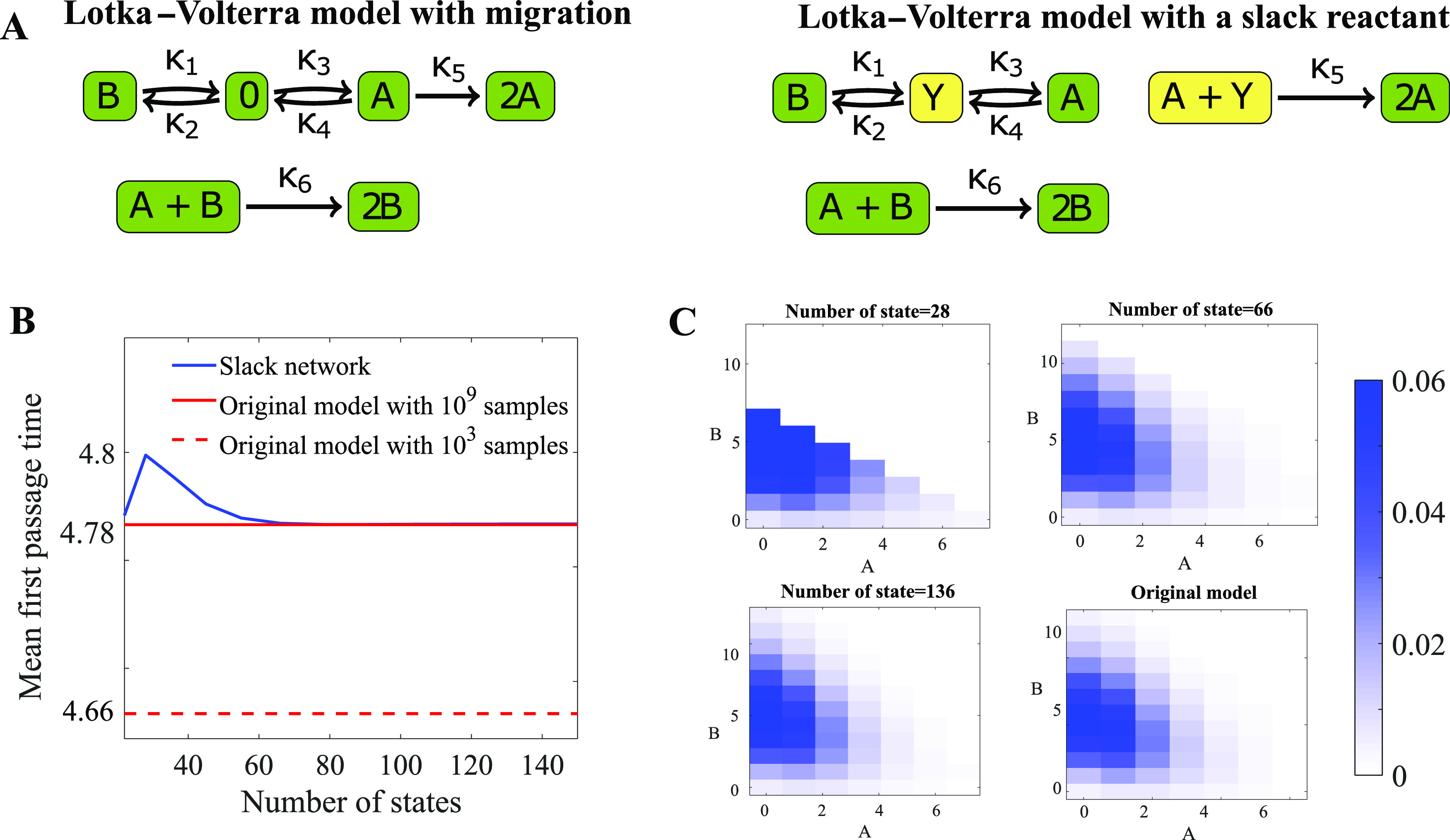
Calculating mean time to extinction of a Lotka–Volterra model with migration using slack reactants. (a) The reaction network for the Lotka–Volterra model with migration (left). The Lotka–Volterra model with a slack reactant *Y* (right). The parameters are *κ*_1_ = 0.1, *κ*_2_ = 0.1, *κ*_3_ = 0.2, *κ*_4_ = 0.6, *κ*_5_ = 0.2, and *κ*_6_ = 0.2. (b) Convergence of the mean time to extinction of the slack network (blue) to the true network (solid red). (c) Heatmaps of the probability density at time 1000 of the original model and the slack system with various truncation size (see Sec. VII A for details).

Consider a Lotka–Volterra model with migration shown in Fig. 4(a). In this model, species *A* is the prey, and species *B* is the predator. Clearly, the state space of this model is infinite (*A*, *B*) such that *A* ≥ 0, *B* ≥ 0. We will use slack reactants to determine the expected time to extinction of either species. More specifically, let *K* = {(*A*, *B*) : *A* = 0 or *B* = 0}. We will calculate the mean first arrival time to *K* from an initial condition [*A*(0), *B*(0)]. [In our simulations in Fig. 4, we chose (*A*(0), *B*(0)) = (3, 3).]

To generate our slack network, we choose a conservation bound *w* · (*A*, *B*)^⊤^ ≤ *N* with *w* = (1, 1). As we discussed in Sec. III C, this *w* minimizes the intrusiveness of slack reactants because the number of reactions *ν* → *ν*′ such that (*ν*′ − *ν*) · *w* > 0 is minimized. By using the algorithm introduced in Sec. III B, we generate a regular slack network (21) with a slack reactant *Y*,
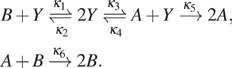
(21)As the slack reactant *Y* in reactions *B* + *Y* ⇌ 2*Y* ⇌ *A* + *Y* can be canceled, we further generate the optimized slack network shown in Fig. 4(a). We let *A*(0) + *B*(0) + *Y*(0) = *N*, which is the conservation quantity of the new network.

Let *τ* be the first passage time from our initial condition to *K*. First, we examine the accessibility of the set *K*. Because our reaction network contains creation and destruction of all species (i.e., *B* ⇌ 0/ ⇌ *A*), the original model is irreducible and any state is accessible to *K*. Furthermore, Theorem VI.1 guarantees that the stochastic model associated with the optimized slack network is also accessible to *K* from any state.

Next, by showing there exists a Lyapunov function satisfying the condition of Theorem V.4 for the original model, we are guaranteed that the first passage times from our slack network will converge to the true first passage times (see Appendix E 1 for more details). Therefore, as the plot shows in Fig. 4(b), the mean first extinction time of the slack network converges to that of the original model as *N* increases. The mean first passage time of the original model was obtained by averaging 10^9^ sample trajectories. These trajectories were computed serially on a commodity machine and took 4.6 h to run. In contrast, the mean first passage times of the slack systems were computed analytically on the same computer and took at most 13 s. Figure 4 also shows that using only 10^3^ samples is misleading as the simulation average has not yet converged to the true mean first passage time. Finally, as expected from Theorem V.1, the probability density of the slack network converges to that of the original network [see Fig. 4(c)].

### B. Protein synthesis with a slow toggle switch

**FIG. 5.**
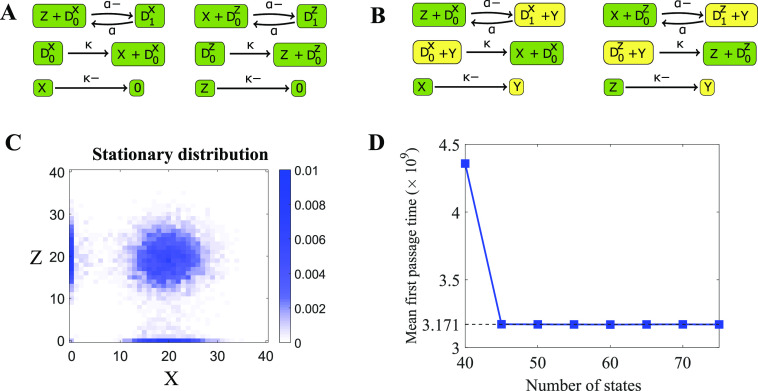
**Calculating mean first passage time of a slow toggle switch using slack reactants.** (a) Protein synthesis with slow toggle switch. (b) The toggle switch model with a slack reactant *Y*. Parameters are *α*− = 0.1, *α* = 10, *κ* = 2000, and *κ*− = 100. (c) Multimodal long-term probability distribution of the original model. (d) Convergence of the first passage time of the slack system with various truncation sizes (see Sec. VII B for details).

We now consider a protein synthesis model with a toggle switch [see Fig. 5(a)]. Protein species *X* and *Z* may be created but only when their respective genes *D*^*X*^ or *D*^*Z*^ are in the active (unoccupied) state, $D_{0}^{X}$ and $D_{0}^{Z}$. Each protein acts as a repressor for the other by binding at the promoter of the opposite gene and forcing it into the inactive (occupied) state ($D_{1}^{X}$ and $D_{1}^{Z}$). In this system, we consider only one copy of each gene so that $D_{0}^{X}+D_{1}^{X}=D_{0}^{Z}+D_{1}^{Z}=1$ for all time. Thus, we focus primarily on the state space of protein numbers only (*X*, *Z*).

The deterministic form of such systems is often referred to as a “bi-stable switch” as it is characterized by steady states (X^*^, 0) (*X* “on” and *Z* “off”) and (0, Z^*^) (*X* “off” and *Z* “on”). This stochastic form of toggle switch has been shown to exhibit a multi-modal long-term probability density due to switches between these two deterministic states due to rapid significant changes in the numbers of proteins *X* and *Z* by synthesis or degradation (depending on the state of promoters).^34^ Figure 5(c) shows that the associated stochastic system admits a *tri-modal* long-term probability density. Thus, the system transitions from one mode to other modes and, for the kinetic parameters chosen in Fig. 5, rarely leaves the region *R* = {(*X*, *Z*)|0 ≤ *X* ≤ 30 or 0 ≤ *Z* ≤ 30}. Significant departures from a stable region of a genetic switch may be associated with irregular and diseased cell fates. As such, the first passage time of this system outside of *R* may indicate the appearance of an unexpected phenotype in a population. Because this event is rare, estimating first passage times with direct stochastic simulations, such as with the Gillespie algorithm,^6^ will be complicated by the long time taken to exit the region.

As in the previous example, slack systems provide a valuable tool for direct calculation of mean first passage times. In this example, we consider the time a trajectory beginning at state $\left(X,Z,D_{0}^{X},D_{0}^{Z}\right)=\left(0,0,1,1\right)$ enters the target set *K* = {(*X*, *Z*)|*X* > 30 and *Z* > 30} = *R*^*c*^ and compute *τ*, the first passage time to *K*.

Since the species corresponding to the status of promoters ($D_{0}^{X},D_{1}^{X},D_{0}^{Z}$ and $D_{1}^{Z}$) are already bounded, we use the conservation bound *X* + *Z* ≤ *N* to generate a regular slack network in Fig. 5(b) with the algorithm introduced in Sec. III B. The original toggle switch model is irreducible (because of the degradation *X* → 0, *Z* → 0 and protein synthesis $Z+D_{0}^{X}\leftarrow D_{1}^{X}$, $X+D_{0}^{Z}\leftarrow D_{1}^{Z}$ reactions). Moreover, by Theorem VI.1, the degradation reactions guarantee that the slack system is also accessible to *K* from any state.

As shown in Fig. 5(d), the mean first passage time of the slack system appears to be converging to approximately 3.171 × 10^9^. To prove that the limit of the slack system is actually the original mean first passage time, we construct a Lyapunov function satisfying the conditions of Theorem V.4. See Appendix E 2 for more details about the construction of the Lyapunov function.

### C. The exclusive mutual inhibition, self-activation model

The exclusive Mutual Inhibition, Self-Activation (ExMISA) model is a cell-fate decision model that contains two genes *A* and *B*, each of which produces transcription factors *X* and *Z*.^35,36^ The transcription factors each bind to their own gene, promoting their own production, as well as binding to the other gene, inhibit the production of the other transcription factor. We consider the ExMISA model described by the following reaction network:
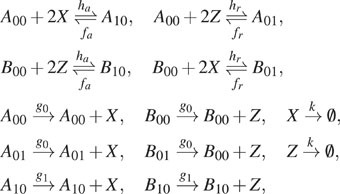
(22)where *g*_0_ < *g*_1_ so that the rate of production of *X* and *Z* is highest when genes *A* and *B* are in the “on-state,” *A*_10_ and *B*_10_, respectively. The transcription factor binding/unbinding rates *f*_*a*_, *h*_*a*_, *f*_*r*_, and *h*_*r*_ are much smaller than the rates of protein production/degradation. (In our simulations, we follow the previous paper^36^ and set *f*_*a*_ = 10^−5^, *f*_*r*_ = 1, *h*_*a*_ = 10^−5^, *h*_*r*_ = 10^−1^, *g*_0_ = 4, *g*_1_ = 18, and *k* = 1.) Hence, the gene state switching reactions are operating in the slow-time scale, while the protein production/degradation reactions are operating in the fast-time scale.

**FIG. 6.**
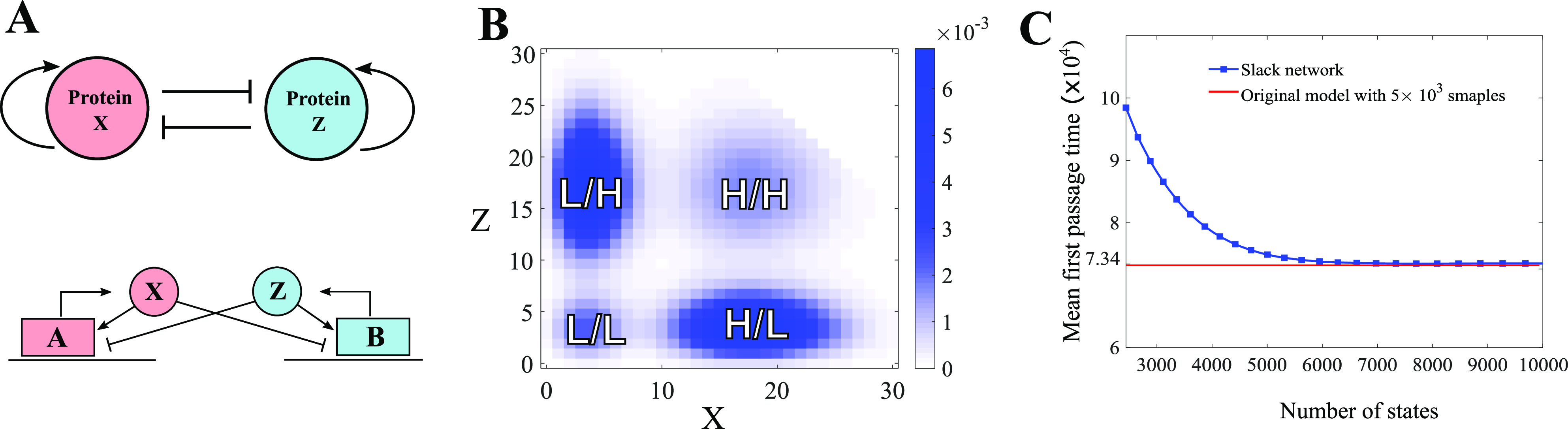
Calculating mean first passage time of ExMISA. (a) Schematic description of ExMISA. (b) Multimodal long-term probability distribution of the ExMISA model with the slack network (23). The modes are labeled by status (H = high, L = low). (c) Approximating the mean transition time from the low/high mode to the high/low mode under a slack system with different truncation size (blue curve) and by time averages from 5000 direct Gillespie simulations (red line) (see Sec. VII C for more details).

Similar to the toggle switch model we introduced in Sec. VII B,^35^ these distinct scales create multiple modes in the long-term system behavior. However, because *X* and *Z* can still be produced when *A* and *B* are in the “off-state,” the behavior of the ExMISA model is more complicated. The system admits four modes in the long-term probability landscape, each of which corresponds to a phenotype of cell [see Fig. 6(b)]. Once the system settles down at one mode, it is extremely rare to transition to other mode because of the slow-time scale.^36,37^

Margaret *et al.*^36^ used a rare-event based simulation method to study metastable gene regulatory systems, including ExMISA, and approximated the long-time probability landscape and time between phenotype-transitions.

In this section, we study the ExMISA model with slack reactants. First, we construct the slack network in (23) with the slack reactant *Y*. We then use the new network to estimate long-term probability distribution and approximate the mean first transition time between two phenotype modes of the original ExMISA model. Note that the probability density can be explicitly derived for a Markov chain with a finite-state space as *p*(*t*) = *μe*^*Qt*^, where *Q* is the transition rate matrix and *μ* is the initial distribution. Hence, we compute the long-time probability density function for the slack network (23) [Fig. 6(b)]. The four peaks in the probability density correspond to high/high, high/low, low/high, and low/low statuses of *X*/*Z*. Then, we estimate the mean transition time from the low/high status to high/low status and show the convergence of the mean transition time in Fig. 6(c). (More specifically, we begin a trajectory at the state *X* = 5, *Z* = 15, *A*_00_ = 0, *A*_01_ = 1, *A*_10_ = 0, *B*_00_ = 0, *B*_01_ = 0, *B*_10_ = 1 and calculate the time it reaches the unit ball centered at *X* = 15, *Z* = 5.) The mean first passage time of the original ExMISA was estimated by averaging 5 × 10^3^ sample trajectories obtained using the Gillespie algorithm. A commodity machine was used to simulate these trajectories in parallel (parfor in Matlab with six workers) and took 3.52 h to run. In contrast, on the same computer, our slack system (23) took only 6 min to compute the mean first passage time for each conservation quantity *N*,
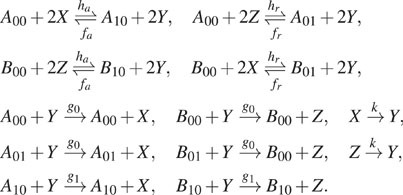
(23)

## VIII. DISCUSSION AND CONCLUSIONS

We propose a new state space truncation method for stochastic reaction networks (see Sec. III). In contrast to other methods, such as FSP, sFSP, and finite buffer methods, we truncate the state space indirectly by expanding the original chemical reaction network to include slack reactants. The truncation is imposed through user-defined conservation relations among species. The explicit network structure of slack reactants facilitates proofs of convergence (Sec. V) and allows the use of existing software packages to study the slack network itself.^17^ Indeed, any user-defined choices for conservation laws, conservation amounts, and stoichiometric structure can be used to construct a slack network with our algorithm. We provide guidelines for optimal user choices that can increase the similarity between the slack system and the original model (see Sec. III C).

Slack systems can be used to estimate the dynamical behavior of the original stochastic model. In Sec. IV, we used a simple example to show that the slack method can lead to a better approximation for the mean first passage time than the sFSP method and the finite buffer method. In particular, in Sec. V, we provide theorems that show that the slack system approximates the probability density and the mean first passage time of the original system. Because slack networks preserve network properties, such as weak reversibility, the slack system is also likely to have the same accessibility to a target state as the original model (see Sec. VI). In particular, we note that weak reversibility guarantees that our slack truncation does not introduce absorbing states.

In Sec. VI, we show that this truncation method is natural in the sense that the stationary distributions of the original and slack systems are identical up to multiplication by a constant when the original system is complex balanced. Finally, in Sec. VII, we use slack networks to calculate first passage times for two biological examples. Our method can be useful to study various biological systems for which we estimate rare event probabilities and mean first passage times. We expect that this new theoretical framework for state space truncation will be useful in the study of other biologically motivated stochastic chemical reaction systems.

## AUTHORS’ CONTRIBUTIONS

G.E. and S.S. contributed equally to this work.

## DATA AVAILABILITY

The data that support the findings of this study are available from the corresponding author upon reasonable request.

## APPENDIX A: FIRST PASSAGE TIME FOR MARKOV PROCESSES WITH FINITELY MANY STATES

The Markov chain associated with a slack network has always a finite state space. There are many different methods to analytically derive the mean first passage time of a Markov chain with a finite state space.^22,38,39^ In this paper, we use the method of Laplace transform, which is also used in Ref. 39.

For a continuous time Markov process defined on a finite state space S, let *Q* be the transition rate matrix, i.e., *Q*_*ij*_ is the transition rate from state *i* to state *j* if *i* ≠ *j* and *Q*_*ii*_ = −∑_*j*≠*i*_*Q*_*ij*_.

For a subset $K=\left\{i_{1},i_{2},\ldots ,i_{k}\right\}\subset S$, we define an *absorbing transition matrix Q*_*K*_ that is obtained by removing the *i*_*j*_th row and column from *Q* for *j* = 1, 2, …, *k*. Then, the mean first passage time to the set *K* starting from the *i*th state is the *i*th entry of $-Q_{K}^{-1}1$, where **1** is a column vector with each entry 1.

## APPENDIX B: PROOFS OF CONVERGENCE THEOREMS

In this section, we provide the proofs of the theorems introduced in Sec. V. We use the same notations and the same assumptions as we used in Sec. V. We also use a projection function *q* such that

$$q\left(x_{1},\ldots ,x_{d},y_{1},\ldots ,y_{r}\right)=\left(x_{1},\ldots ,x_{d}\right).$$ (B1)

This linear function *q* projects a complex in a slack network onto a complex in the original network as $q\left(\tilde{\nu }\right)=\nu$ if $\tilde{\nu }$ is obtained by adding slack reactants to *ν*. In the same sense, $q\left({\tilde{\nu }}^{\prime }-\tilde{\nu }\right)=\nu^{\prime }-\nu$ if a reaction $\tilde{\nu }\to {\tilde{\nu }}^{\prime }$ is obtained from a reaction *ν* → *ν*′ by adding slack reactants.

Proof of Theorem V.1. We employ the main idea shown in the proof of Theorem 2.1 in Ref. 25. Let a state **x** and time *t* be fixed. We consider large enough *N* so that $x\in S_{N-1}$. We use an FSP model on $S_{N-1}$ of the original system *X* with the designated absorbing state $x^{*}\in S_{N-1}^{c}$. Let $p_{N-1}^{\text{FSP}}$ be the probability density function of this FSP model. Let *T*_*N*_ be the first time for **X**^*N*^ to hit $S_{N}\backslash S_{N-1}$. We generate a coupling of **X**^*N*^ and the FSP model restricted on $S_{N-1}$ as they move together by *T*_*N*_ and they move independently after *T*_*N*_. Then, $p_{N-1}^{\text{FSP}}\left(x,t\right)=P\left(X^{N}\left(t\right)=x,T_{N}>t\right)$ for $x\in S_{N-1}$ because the FSP model has stayed in $S_{N-1}$ if and only if **X**^*N*^ has never touched $S_{N}\backslash S_{N-1}$. Thus,

$$\begin{array}{ll} p_{N}\left(x,t\right) & =P\left(X^{N}\left(t\right)=x,t<T_{N}\right)+P\left(X^{N}\left(t\right)=x,t\geq T_{N}\right)\\ & =p_{N-1}^{\text{FSP}}\left(x,t\right)+P\left(X^{N}\left(t\right)=x,t\geq T_{N}\right)\\ & \geq p_{N-1}^{\text{FSP}}\left(x,t\right). \end{array}$$ (B2)

Furthermore,

$$\begin{array}{ll} P\left(\right.X^{N}\left(t\right) & =x,t\geq T_{N})\leq P\left(t\geq T_{N}\right)\\ & =p_{N-1}^{\text{FSP}}\left(x^{*},t\right)=1-p_{N-1}^{\text{FSP}}\left(S_{N-1},t\right), \end{array}$$

where we used the fact that after *T*_*N*_, the FSP process is absorbed at **x**^*^. Thus,

$$p_{N-1}^{\text{FSP}}\left(x,t\right)\leq p_{N}\left(x,t\right)\leq p_{N-1}^{\text{FSP}}\left(x,t\right)+1-p_{N-1}^{\text{FSP}}\left(S_{N-1},t\right),$$

and hence,

$$|p_{N}\left(x,t\right)-p_{N-1}^{\text{FSP}}\left(x,t\right)|\leq 1-p_{N-1}^{\text{FSP}}\left(S_{N-1},t\right).$$ (B3)

Note that

$$p_{N-1}^{\text{FSP}}\left(x,t\right)=P\left(X\left(t\right)=x,t<T_{N}\right)\leq p\left(x,t\right).$$

Since *T*_*N*_ increases to ∞ almost surely as *N* increases, $p_{N-1}^{\text{FSP}}\left(x,t\right)$ monotonically increases in *N* and converges to *p*(**x**, *t*) as *N* → ∞ for each $x\in S_{N}$. Then, by using the monotone convergence theorem, the term

$$p_{N-1}^{\text{FSP}}\left(S_{N-1},t\right)=\underset{x\in S}{\sum }p_{N-1}^{\text{FSP}}\left(x,t\right)1_{\left\{x\in S_{N-1}\right\}}$$

converges to p(S,t)=1. Since for any *t* ∈ [0, *T*]

$$\begin{array}{ll} p_{N-1}^{\text{FSP}}\left(S_{N-1},T\right) & =P\left(X^{N}\left(t\right)\in S_{N-1},T<T_{N}\right)\\ & \leq P\left(X^{N}\left(t\right)\in S_{N-1},t<T_{N}\right)=p_{N-1}^{\text{FSP}}\left(S_{N-1},t\right), \end{array}$$

therefore, by taking $\underset{N\to \infty }{\lim}\underset{t\in \left[0,T\right]}{\sup}$ in both sides of (B3), the result follows.□

Proof of Theorem V.2. This proof is a slight generalization of the proof of Theorem 3.3 of Ref. 25. Since the convergence of ‖*p*_*N*_(·, *t*) − *π*_*N*_‖_1_ is independent of *N*, for any *ε* > 0, we choose sufficiently large *t*_0_ such that

$$\|p\left(\cdot ,t_{0}\right)-\pi \left(t_{0}\right){\|}_{1}\leq \varepsilon \text{ and }\|p_{N}\left(\cdot ,t_{0}\right)-\pi_{N}\left(t_{0}\right){\|}_{1}\leq \varepsilon$$

for all *N*. Then, by using the triangle inequalities,

$$\begin{array}{ll} \|\pi \;-\;\pi_{N}{\|}_{1} & \leq \|p\left(\cdot ,t_{0}\right)\;-\;\pi {\|}_{1}\;+\;\|p_{N}\left(\cdot ,t_{0}\right)\;-\;\pi_{N}{\|}_{1}\;+\;\|p\left(\cdot ,t_{0}\right)\;-\;p_{N}\left(\cdot ,t_{0}\right){\|}_{1}\\ & \leq \|p\left(\cdot ,t_{0}\right)-\pi {\|}_{1}+\|p_{N}\left(\cdot ,t_{0}\right)-\pi_{N}{\|}_{1}\\ & \;+\;\|p\left(\cdot ,t_{0}\right)-p_{N-1}^{\text{FSP}}\left(\cdot ,t_{0}\right){\|}_{1}+\|p_{N-1}^{\text{FSP}}\left(\cdot ,t_{0}\right)-p_{N}\left(\cdot ,t_{0}\right){\|}_{1}\\ & \leq 2\varepsilon +\underset{x\in S}{\sum }|p\left(x,t_{0}\right)-p_{N-1}^{\text{FSP}}\left(x,t_{0}\right)|\\ & \;+\;\underset{x\in S}{\sum }|p_{N-1}^{\text{FSP}}\left(x,t_{0}\right)-p_{N}\left(x,t_{0}\right)|. \end{array}$$ (B4)

Note that as we mentioned in the proof of Theorem V.1, we have monotone convergence of $p_{N-1}^{\text{FSP}}\left(x,t_{0}\right)$ to *p*(**x**, *t*_0_) for each $x\in S_{N-1}$ as *N* → ∞. Hence, by the monotone convergence theorem, the first summation in (B4) goes to zero as *N* → ∞. Note further that from (B2), we have

$$|p_{N-1}^{\text{FSP}}\left(x,t_{0}\right)-p_{N}\left(x,t_{0}\right)|=P\left(X^{N}\left(t\right)=x,T_{N}\leq t_{0}\right).$$

Hence, the second summation in (B4) satisfies

$$\begin{array}{ll} \underset{x\in S}{\sum }|p_{N-1}^{\text{FSP}}\left(x,t_{0}\right)-p_{N}\left(x,t_{0}\right)| & \leq \underset{x\in S_{N-1}}{\sum }|p_{N-1}^{\text{FSP}}\left(x,t_{0}\right)-p_{N}\left(x,t_{0}\right)|\\ & \;+\;p_{N-1}^{\text{FSP}}\left(x^{*},t_{0}\right)\\ & \;+\;P\left(X^{N}\left(t\right)\in S_{N}\backslash S_{N-1}\right). \end{array}$$

Note that by (B2),

$$\begin{array}{ll} & \underset{x\in S_{N-1}}{\sum }|p_{N-1}^{\text{FSP}}\left(x,t_{0}\right)-p_{N}\left(x,t_{0}\right)|\\ & \;=\underset{x\in S_{N-1}}{\sum }P\left(X^{N}\left(t_{0}\right)=x,t_{0}\geq T_{N}\right)=P\left(t_{0}\geq T_{N}\right). \end{array}$$

Furthermore, $p_{N-1}^{\text{FSP}}\left(x^{*},t_{0}\right)=P\left(t_{0}\geq T_{N}\right)$ and $P\left(X^{N}\left(t\right)\in S_{N}\backslash S_{N-1}\right)=P\left(T_{N}<t_{0}\right)$. Hence, $\sum_{x\in S}|p_{N-1}^{\text{FSP}}\left(x,t_{0}\right)-p_{N}\left(x,t_{0}\right)|\to 0$ as *N* → ∞ because *T*_*N*_ → ∞ almost surely as *N* → ∞. Consequently, we have

$$\underset{N\to \infty }{\lim}\|\pi -\pi_{N}{\|}_{1}\leq 2\varepsilon .$$

Since we choose an arbitrary *ε*, this completes the proof. In order to prove Theorem V.3, we consider an “absorbing” Markov process associated with *X* and **X**^*N*^. Let $\bar{X}$ and ${\bar{X}}^{N}$ be Markov processes such that

$$\bar{X}\left(t\right)=\left\{\begin{array}{cc} X\left(t\right)\; & \text{if}t<\tau \\ X\left(\tau \right)\; & \text{if}t\geq \tau , \end{array}\right.$$

$${\bar{X}}^{N}\left(t\right)=\left\{\begin{array}{cc} X^{N}\left(t\right)\; & \text{if}t<\tau_{N}\\ X^{N}\left(\tau_{N}\right)\; & \text{if}t\geq \tau_{N}. \end{array}\right.$$

That is, $\bar{X}$ and ${\bar{X}}^{N}$ are coupled processes to **X** and **X**^*N*^, respectively. Furthermore, they are absorbed to *K* once the coupled process (**X** for $\bar{X}$ and **X**^*N*^ for ${\bar{X}}^{N}$) visits the set *K*. These coupled processes have the following relation.

Lemma B.1.
*Let t* ≥ 0 *be fixed and ε* > 0 *be arbitrary. Suppose X admits a stationary distribution π. Suppose further that* $\underset{N\to \infty }{\lim}\|\pi -\pi_{N}{\|}_{1}=0$*. Then, there exists a finite subset* $\bar{K}$ *such that* $P\left(\bar{X}\left(t\right)\in {\bar{K}}^{c}\right)<\varepsilon$ *and* $P\left({\bar{X}}^{N}\left(t\right)\in {\bar{K}}^{c}\right)<\varepsilon$ *for sufficiently large N*.

Proof.
Since the probabilities of $\bar{X}$ and ${\bar{X}}^{N}$ are leaking to *K*, we have for each *t* and for each **x** ∈ *K*^*c*^

$$\begin{array}{ccc} P\left(\bar{X}\left(t\right)=x\right)\leq P\left(X\left(t\right)=x\right)\text{ and }P\left({\bar{X}}^{N}\left(t\right)=x\right)<P\left(X^{N}\left(t\right)=x\right). & & \end{array}$$ (B5)

This can be formally proved as for each *x* ∈ *K*^*c*^,

$$\begin{array}{ll} P\left(X\left(t\right)=x\right) & =P\left(X\left(t\right)=x,\tau >t\right)+P\left(X\left(t\right)=x,\tau \leq t\right)\\ & =P\left(\bar{X}\left(t\right)=x\right)+P\left(X\left(t\right)=x,\tau^{K}\leq t\right)\\ & \geq P\left(\bar{X}\left(t\right)=x\right). \end{array}$$

In the same way, we can prove that $P\left({\bar{X}}^{N}\left(t\right)=x\right)<P\left(X^{N}\left(t\right)=x\right)$ for each **x** ∈ *K*^*c*^. Let *ε*′ > 0 be arbitrary. Then, $\underset{N\to \infty }{\lim}\|\pi -\pi_{N}{\|}_{1}=0$ implies that for any subset *U*, we have

$$\pi \left(U\right)-\varepsilon^{\prime }\leq \pi_{N}\left(U\right)\leq \pi \left(U\right)+\varepsilon^{\prime }$$ (B6)

for sufficiently large *N*. We use this property and (B5) combined with the stationarity of the systems. First, note that we are assuming **X**(0) = **X**^*N*^(0) = **x**_0_ for a fixed **x**_0_. Then, by the definition of the stationary distribution *π*,

$$P_{x_{0}}\left(X\left(t\right)=x\right)\pi \left(x_{0}\right)\leq \underset{z\in S}{\sum }P\left(X\left(t\right)=x|X\left(t\right)=z\right)\pi \left(z\right)=\pi \left(x\right)$$

for each *t* ≥ 0. Then, it follows that

$$P_{x_{0}}\left(X\left(t\right)=x\right)\leq \frac{\pi \left(x\right)}{\pi \left(x_{0}\right)}=\gamma_{1}\pi \left(x\right)\text{ for any}x\text{and}t,$$ (B7)

where *γ*_1_ = 1/*π*(**x**_0_). In the same way, for any *N*, it follows that

$$P_{x_{0}}\left(X^{N}\left(t\right)=x\right)\leq \frac{\pi_{N}\left(x\right)}{\pi_{N}\left(x_{0}\right)}\leq \gamma_{2}\pi_{N}\left(x\right)\text{ for any}x\text{and}t,$$ (B8)

where *γ*_2_ = 1/(*π*(**x**_0_) − *ε*′) ≥ 1/*π*_*N*_(**x**_0_) by (B6) with sufficiently small *ε*′. Second, there exists a finite set $\bar{K}$ such that $\pi \left({\bar{K}}^{c}\right)<\varepsilon^{\prime }$ because *π* is a probability distribution. Furthermore, by (B6), $\pi_{N}\left({\bar{K}}^{c}\right)\leq \pi \left({\bar{K}}^{c}\right)+\varepsilon^{\prime }<2\varepsilon^{\prime }$ for any sufficiently large *N*. Finally, we choose $\varepsilon^{\prime }=\frac{\varepsilon }{2\gamma }$, where *γ* = max{*γ*_1_, *γ*_2_}. Then, by summing up (B5), (B7), and (B8) over $x\in {\bar{K}}^{c}$, the result follows. We prove Theorem V.3 by using the “absorbing” Markov processes $\bar{X}$ and ${\bar{X}}^{N}$ coupled to **X** and **X**^*N*^, respectively.

Proof of Theorem V.3. We first break the term *P*(*τ* > *t*) − *P*(*τ*_*N*_ > *t*) to show that

$$\underset{N\to \infty }{\lim}\underset{t\in \left[0,T\right]}{\sup}|P\left(\tau >t\right)-P\left(\tau_{N}>t\right)|=0.$$

Let *ε* > 0 be an arbitrarily small number. Let $\bar{K}$ be a finite set we found in Lemma B.1. Then, by using triangular inequalities,

$$\begin{array}{ll} \left|P\left(\tau >t\right)-P\left(\tau_{N}>t\right)\right| & =|P\left(\bar{X}\left(t\right)\in K^{c}\right)-P\left({\bar{X}}^{N}\left(t\right)\in K^{c}\right)|\\ & \leq \underset{x\in K^{c}\cap \bar{K}}{\sum }|P\left(\bar{X}\left(t\right)=x\right)-P\left({\bar{X}}^{N}\left(t\right)=x\right)|\\ & \;+\underset{x\in K^{c}\cap {\bar{K}}^{c}}{\sum }|P\left(\bar{X}\left(t\right)=x\right)-P\left({\bar{X}}^{N}\left(t\right)=x\right)|\\ & \leq \underset{x\in K^{c}\cap \bar{K}}{\sum }|P\left(\bar{X}\left(t\right)=x\right)-P\left({\bar{X}}^{N}\left(t\right)=x\right)|\\ & \;+P\left(\bar{X}\left(t\right)\in {\bar{K}}^{c}\right)+P\left({\bar{X}}^{N}\left(t\right)\in {\bar{K}}^{c}\right)\\ & \leq \underset{x\in K^{c}\cap \bar{K}}{\sum }|P\left(\bar{X}\left(t\right)=x\right)-P\left({\bar{X}}^{N}\left(t\right)=x\right)|+2\varepsilon . \end{array}$$ (B9)

Note that by the same proof of Theorem V.1, we have the convergence

$$\underset{N\to \infty }{\lim}\underset{t\in \left[0,T\right]}{\sup}|P\left(\bar{X}\left(t\right)=x\right)-P\left({\bar{X}}^{N}\left(t\right)=x\right)|=0\text{ for each}x\in S.$$

Since the summation $\sum_{x\in K^{c}\cap \bar{K}}$ is finite, we have that by taking *N* → ∞ in (B9),

$$\underset{N\to \infty }{\lim}\underset{t\in \left[0,T\right]}{\sup}|P\left(\tau^{K}>t\right)-P\left(\tau_{N}^{K}>t\right)|=0+2\varepsilon .$$ (B10)

Now, to show the convergence of the mean first passage times, note that

$$|E\left(\tau \right)-E\left(\tau_{N}\right)|\leq \int_{0}^{\infty }|P\left(\tau >t\right)-P\left(\tau_{N}>t\right)|dt,$$

where the integrand is bounded by *P*(*τ*_*K*_ > *t*) + *g*(*t*). Condition (iii) in Theorem V.3 implies that *E*(*τ*_*k*_) < ∞ and $\int_{0}^{\infty }g\left(t\right)dt<\infty$. Hence, the dominant convergence theorem and (B10) imply that

$$\underset{N\to \infty }{\lim}|E\left(\tau \right)-E\left(\tau_{N}\right)|=0.$$

□

The existence of a special Lyapunov function ensures that the conditions in Theorem V.3 hold. In order to use the absorbing Markov processes, as we did in the previous proof, we define a Lyapunov function for $\bar{X}$ based on a given Lyapunov function *V*. Let ${\bar{\lambda }}_{\nu \to \nu^{\prime }}$ and ${\bar{\lambda }}_{\nu \to \nu^{\prime }}^{N}$ denote the intensity of a reaction *ν* → *ν*′ for $\bar{X}$ and ${\bar{X}}^{N}$, respectively. For a given function *V* such that *V*(**x**) ≥ 1 for any x∈S, we define $\bar{V}$ such that

$$\bar{V}\left(x\right)=\left\{\begin{array}{cc} V\left(x\right)\; & \text{if}x\in K^{c}\\ 1\; & \text{if}x\in K \end{array}\right.$$

so that $V\left(x\right)\geq \bar{V}\left(x\right)$ for any **x**. Note that ${\bar{\lambda }}_{\nu \to \nu^{\prime }}\left(x\right)=\lambda_{\nu \to \nu^{\prime }}\left(x\right)$ if **x** ∈ *K*^*c*^. Hence, for each **x** ∈ *K*^*c*^,

$$\begin{array}{ll} & \underset{\nu \to \nu^{\prime }\in R}{\sum }{\bar{\lambda }}_{\nu \to \nu^{\prime }}\left(x\right)\left(\bar{V}\left(x+\nu^{\prime }-\nu \right)-\bar{V}\left(x\right)\right)\\ & \;\leq \underset{\nu \to \nu^{\prime }\in R}{\sum }\lambda_{\nu \to \nu^{\prime }}\left(x\right)\left(V\left(x+\nu^{\prime }-\nu \right)-V\left(x\right)\right) \end{array}$$ (B11)

because $V\left(x\right)=\bar{V}\left(x\right)$ and $V\left(x+\nu^{\prime }-\nu \right)\geq \bar{V}\left(x+\nu^{\prime }-\nu \right)$ for every reaction **x** + *ν*′ − *ν*. Moreover, ${\bar{\lambda }}_{\nu \to \nu^{\prime }}\left(x\right)=0\leq \lambda_{\nu \to \nu^{\prime }}\left(x\right)$ if **x** ∈ *K* by the definition of $\bar{X}$ for each reaction *ν* → *ν*′. Hence, for each **x** ∈ *K*,

$$\underset{\nu \to \nu^{\prime }\in R}{\sum }{\bar{\lambda }}_{\nu \to \nu^{\prime }}\left(x\right)\left(\bar{V}\left(x+\nu^{\prime }-\nu \right)-\bar{V}\left(x\right)\right)=0.$$ (B12)

From (B11) and (B12), therefore, we conclude that for any **x**,

$$\begin{array}{ll} & \underset{\nu \to \nu^{\prime }\in R}{\sum }{\bar{\lambda }}_{\nu \to \nu^{\prime }}\left(x\right)\left(\bar{V}\left(x+\nu^{\prime }-\nu \right)-\bar{V}\left(x\right)\right)\\ & \;\leq \left\{\begin{array}{cc} -CV\left(x\right)+D\; & \text{if}x\in K^{c}\\ 0\; & \text{if}x\in K \end{array}\right.\\ & \;\leq -C^{\prime }\bar{V}\left(x\right)+D^{\prime }, \end{array}$$ (B13)

where *C*′ = *C* and *D*′ = max{*C* + 1, *D*}.

Proof of Theorem V.4. In this proof, we show that all the conditions in Theorem V.3 are met by using the given function *V*. First, we show that *X* admits a stationary distribution. This follows straightforwardly by Theorem 3.2 of Ref. 25 because condition 3 in Theorem V.4 means that *V* is a Lyapunov function for **X**. The condition basically means that every outward reaction *ν* → *ν*′ (i.e., $x+\nu^{\prime }-\nu \in S_{N}^{c}$ and $x\in S_{N}$) in R gives a non-negative drift as *V*(**x** + *ν*′ − *ν*) − *V*(**x**) ≥ 0. We use condition 2 in Theorem V.4 to show that *V* is also a Lyapunov function for **X**^*N*^ for any N. We denote by $\lambda_{\tilde{\nu }\to {\tilde{\nu }}^{\prime }}^{N}$ the intensity of a reaction $\tilde{\nu }\to {\tilde{\nu }}^{\prime }$ in $\left(\tilde{S},\tilde{C},\tilde{R},\Lambda^{N}\right)$. Suppose that a reaction $\tilde{\nu }\to {\tilde{\nu }}^{\prime }\in \tilde{R}$ is obtained from $\nu \to \nu^{\prime }\in R$ by adding a slack reactant. That is, $q\left({\tilde{\nu }}^{\prime }-\tilde{\nu }\right)=\nu^{\prime }-\nu$, where *q* is the projection function defined at (B1). Then, by the definition (10) of the intensity in a slack network, we have $\lambda_{\tilde{\nu }\to {\tilde{\nu }}^{\prime }}^{N}\left(x\right)=0$ when $x+q\left({\tilde{\nu }}^{\prime }-\tilde{\nu }\right)/\in S_{N}$ because the **X**^*N*^ is confined in $S_{N}$. Furthermore, by condition 2 in Theorem V.4, *V*(**x** + *ν*′ − *ν*) ≥ *V*(*x*) when $x+q\left({\tilde{\nu }}^{\prime }-\tilde{\nu }\right)/\in S_{N}$ because this means that $x+\nu^{\prime }-\nu \in S_{M}$ for some *M* > *N*. This implies that if $x+\nu^{\prime }-\nu /\in S_{N}$,

$$\begin{array}{ll} 0 & =\lambda_{\tilde{\nu }\to {\tilde{\nu }}^{\prime }}^{N}\left(x\right)\left(V\left(x+\nu^{\prime }-\nu \right)-V\left(x\right)\right)\\ & \leq \lambda_{\nu \to \nu^{\prime }}\left(x\right)\left(V\left(x+\nu^{\prime }-\nu \right)-V\left(x\right)\right). \end{array}$$ (B14)

In case $x+q\left({\tilde{\nu }}^{\prime }-\tilde{\nu }\right)\in S_{N}\subseteq S$, we have that $\lambda_{\tilde{\nu }\to {\tilde{\nu }}^{\prime }}^{N}\left(x\right)=\lambda_{\nu \to \nu^{\prime }}\left(x\right)$ by definition (10). Hence, by condition 3 and (B14), we have for any **x**

$$\begin{array}{ll} & \underset{\tilde{\nu }\to {\tilde{\nu }}^{\prime }\in R}{\sum }\lambda_{\tilde{\nu }\to {\tilde{\nu }}^{\prime }}^{N}\left(x\right)\left(V\left(x+\nu^{\prime }-\nu \right)-V\left(x\right)\right)\\ & \;\leq \underset{\nu \to \nu^{\prime }\in R}{\sum }\lambda_{\nu \to \nu^{\prime }}\left(x\right)\left(V\left(x+\nu^{\prime }-\nu \right)-V\left(x\right)\right)\\ & \;\leq -CV\left(x\right)+D. \end{array}$$ (B15)

Thus, *V* is also a Lyapunov function for **X**^*N*^. Hence, Theorem 6.1 (the Foster–Lyapunov criterion for exponential ergodicity) in Ref. 26 implies that for each *N*, there exist *β* > 0 and *η* > 0, which are only dependent of *C* and *D*, such that

$$\|p_{N}\left(\cdot ,t\right)-\pi_{N}{\|}_{1}\leq \beta V\left(x_{0}\right)e^{-\eta t}.$$

This guarantees that the condition in Theorem V.2 holds with *h*(*t*) = *βV*(**x**_0_)*e*^−*ηt*^. Hence, we have $\underset{N\to \infty }{\lim}\|\pi -\pi_{N}{\|}_{1}=0$ by Theorem V.2. Now, to show that the first passage time *τ* has the finite mean, we apply the Foster–Lyapunov criterion to $\bar{X}$. Since (B13) meets the conditions of Theorem 6.1 of Ref. 26, the probability of $\bar{X}$ converges in time to its stationary distribution exponentially fast. That is, for any subset *U*, there exist $\bar{\beta }>0$ and $\bar{\eta }>0$ such that

$$\|P\left(\bar{X}\left(t\right)\in U\right)-\bar{\pi }\left(U\right)\|\leq \beta V\left(x_{0}\right)e^{-\eta t},$$

where $\bar{\pi }$ is the stationary distribution of $\bar{X}$. This, in turn, implies that

$$\begin{array}{ll} E\left(\tau_{K}\right) & =\int_{0}^{\infty }P\left(\tau >t\right)dt\\ & =\int_{0}^{\infty }P\left(\bar{X}\left(t\right)\in K^{c}\right)dt\\ & \leq \int_{0}^{\infty }|P\left(\bar{X}\left(t\right)\in K^{c}\right)-\bar{\pi }\left(K^{c}\right)|+\bar{\pi }\left(K^{c}\right)dt\\ & \leq \int_{0}^{\infty }\beta V\left(x_{0}\right)e^{-\eta t}dt<\infty , \end{array}$$ (B16)

where the second inequality follows as $\bar{\pi }\left(K^{C}\right)=0$, which is because $\bar{X}$ is eventually absorbed in *K* as the original process **X** is irreducible and closed. Finally, we show that for any *N*, there exists *g*(*t*) such that *P*(*τ*_*N*_ < *t*) < *g*(*t*) and $\int_{0}^{\infty }g\left(t\right)dt<\infty$ for the first passage time *τ*_*K*,*N*_. By the same reasoning we used to derive (B13), we also derive by using (B15) that

$$\underset{\tilde{\nu }\to {\tilde{\nu }}^{\prime }\in R}{\sum }{\bar{\lambda }}_{\tilde{\nu }\to {\tilde{\nu }}^{\prime }}^{N}\left(x,y\right)\left(\bar{V}\left(x+\nu^{\prime }-\nu \right)-\bar{V}\left(x\right)\right)\leq -C^{\prime }\bar{V}\left(x\right)+D^{\prime }.$$ (B17)

Hence, in the same way as used for (B16), we have the exponential ergodicity of ${\bar{X}}^{N}$ by Theorem 6.1 of Ref. 26, and then, we derive that

$$\begin{array}{ll} P\left(\tau_{N}>t\right) & =P\left({\bar{X}}^{N}\left(t\right)\in K^{c}\right)\\ & \leq |P\left(\bar{X}\left(t\right)\in K^{c}\right)-{\bar{\pi }}_{N}\left(K^{c}\right)|+{\bar{\pi }}_{N}\left(K^{c}\right)\\ & \leq \beta V\left(x_{0}\right)e^{-\eta t}, \end{array}$$

where ${\bar{\pi }}_{N}$ is the stationary distribution of ${\bar{X}}^{N}$, which also has zero probability in *K*^*C*^ as ${\bar{X}}^{N}$ is eventually absorbed in *K*. Since *β* and *η* only depend on *C*′ and *D*′, we let *g*(*t*) = *βV*(**x**_0_)*e*^−*ηt*^ that satisfies that *P*(*τ*_*K*,*N*_ > *t*) ≤ *g*(*t*) for any *N* and $\int_{0}^{\infty }g\left(t\right)dt<\infty .□$

## APPENDIX C: PROOFS OF ACCESSIBILITY THEOREMS

In this section, we prove Theorem VI.1. We begin with a necessary lemma. In the following lemma, for a given reaction system (S,C,R,Λ), we generate a slack system $\left(\tilde{S},\tilde{C},\tilde{R},\Lambda^{N}\right)$ admitting a single slack reactant *Y*. We denote by **w** the vector for which the slack system admits the conservation bound **w** · **x** ≤ *N* for each state *x* of the slack system. We also let *c* be the maximum stoichiometric coefficient of the slack reactant *Y* in the slack network. Finally, note that, as shown in Sec. III B, $\tilde{R}$ and R have the one-to-one correspondence as every reaction in $\tilde{R}$ is obtained by adding the slack reactant to a reaction in R. For the proof of Theorem V.4, we use the projection function *q* defined at (B1).

Lemma C.1.
*For a reaction system* (S,C,R,Λ)*, let* $\left(\tilde{S},\tilde{C},\tilde{R},\Lambda^{N}\right)$ *be a slack system with a single slack reactant Y and a conservation vector* **w**. *Let c be the maximum stoichiometric coefficient of the slack reactant Y in* $\left(\tilde{S},\tilde{C},\tilde{R},\Lambda^{N}\right)$*. Then, if c* ≤ *N* − (**w** · **x**) *for a state* **x***, then*

$$\lambda_{\tilde{\nu }\to {\tilde{\nu }}^{\prime }}^{N}\left(x\right)>0\text{ if only if }\lambda_{\nu \to \nu^{\prime }}\left(x\right)>0,$$

*where* $\tilde{\nu }\to {\tilde{\nu }}^{\prime }\in \tilde{R}$ *and* $\nu \to \nu^{\prime }\in R$ *are reactions such that* $q\left(\tilde{\nu }\right)=\nu$ *and* $q\left({\tilde{\nu }}^{\prime }\right)=\nu^{\prime }$.

Proof.
Let $\tilde{\nu }\in \tilde{C}$ such that $\tilde{\nu }=\left(\nu ,u_{\nu }\right)$ with the stoichiometric coefficient *u*_*ν*_ of *Y*. By the definition of $\lambda_{\tilde{\nu }\to {\tilde{\nu }}^{\prime }}^{N}$ shown in (10),

$$\lambda_{\tilde{\nu }\to {\tilde{\nu }}^{\prime }}^{N}\left(x\right)>0\text{ if only if }\lambda_{\nu \to \nu^{\prime }}\left(x\right)>0,$$

so long as *y* = *N* − **w** · **x** ≥ *u*_*ν*_. Since $c=\underset{\nu \in C}{\max}\left\{u_{\nu }\right\}$, the result follows.

Proof of Theorem VI.1. We denote by S the irreducible and closed state space of **X** such that **X**(0) = **x**_0_. We also denote by $S_{N}$ the communication class of **X**^*N*^ such that $x_{0}\in S_{N}$ for each *N*. Then, $S_{N}\subseteq S$, and the irreducibility of S guarantees that $\cup_{i=1}^{\infty }S_{i}=S$. Therefore, there exists *N*_1_ such that $S_{N,x_{0}}\cap K\neq 0\text{/}$ if *N* ≥ *N*_1_. This implies that it is enough to show that $S_{N,x_{0}}$ is closed for *N* large enough because $S_{N,x_{0}}$ is a finite subset. To prove this, we claim that there exists *N*_0_ such that if *N* ≥ *N*_0_; then, $S_{N}$ admits no out-going flow. To prove the claim by contradiction, we assume that for a sequence *N*_*k*_ such that lim_*k*→∞_*N*_*k*_ = ∞, there is an out-going flow from $S_{N_{k}}$ to a state $x_{k}\in S_{N_{k}}^{c}$. We find a sequence of reactions with which $X_{N_{k}}$ returns to $S_{N_{k}}$ from **x**_*k*_ for all large enough *k*. First of all, note $\left\{X_{i}\to Y\right\}\subset \tilde{R}$ because each reaction $X_{i}\to 0\text{/}\in R$ is converted to *X*_*i*_ → *Y* in any optimized slack network by its definition of an optimized slack network (see Sec. III C). Hence, for each *k*, by firing the reactions {*X*_*i*_ → *Y*}, $X_{N_{k}}$ can reach 0 = (0, 0, …, 0) from **x**_*k*_. The next aim is to show that for any fixed *k* large enough, there exist a sequence of reactions $\left\{{\tilde{\nu }}_{1}\to {\tilde{\nu }}_{1}^{\prime },\ldots {\tilde{\nu }}_{n}\to {\tilde{\nu }}_{n}^{\prime }\right\}\subset \tilde{R}$ such that we have (i) $\lambda_{{\tilde{\nu }}_{i}\to {\tilde{\nu }}_{i}^{\prime }}^{N_{k}}\left(x_{k}+\sum_{j=1}^{i-1}\left({\tilde{\nu }}_{j}^{\prime }-{\tilde{\nu }}_{j}\right)\right)>0$ for each *i* and (ii) $x_{k}+\sum_{j=1}^{n}\left(q\left({\tilde{\nu }}_{j}^{\prime }-{\tilde{\nu }}_{j}\right)\right)\in S_{N_{k}}$. This means that $X_{N_{k}}$ can reach $S_{N_{k}}$ from **x**_*k*_ along the reactions ${\tilde{\nu }}_{i}\to {\tilde{\nu }}_{i}$ in that order. For this aim, we make use of the irreducibility of S so that there exists a sequence of reactions $\left\{\nu_{1}\to \nu_{1}^{\prime },\ldots ,\nu_{n}\to \nu_{n}^{\prime }\right\}\subset R$ such that (i) $\lambda_{\nu_{i}\to \nu_{i}^{\prime }}\left(0+\sum_{j=1}^{i-1}\left(\nu_{j}^{\prime }-\nu_{j}\right)\right)>0$ for each *i* and (ii) $0+\sum_{j=1}^{n}\left(\nu_{j}^{\prime }-\nu_{j}\right)\in S_{N_{1}}$. By making *N*_0_ ≥ *N*_1_ large enough, we have that if *N*_*k*_ ≥ *N*_0_, then

$$w\cdot \left(0+\overset{i}{\underset{j=1}{\sum }}\left(\nu_{j}^{\prime }-\nu_{j}\right)\right)<N-c\text{ for each}i.$$

Hence, by Lemma C.1, for each reaction ${\tilde{\nu }}_{i}\to {\tilde{\nu }}_{i}^{\prime }$ such that $q\left({\tilde{\nu }}_{i}\right)=\nu_{i}$ and $q\left({\tilde{\nu }}_{i}^{\prime }\right)=\nu_{i}^{\prime }$, we have

$$\lambda_{{\tilde{\nu }}_{i}\to {\tilde{\nu }}_{i}^{\prime }}^{N}\left(0+\overset{i-1}{\underset{j=1}{\sum }}\left(q\left({\tilde{\nu }}_{j}^{\prime }-{\tilde{\nu }}_{j}\right)\right)\right)>0\text{ for each}i.$$ (C1)

Finally, since $q\left({\tilde{\nu }}_{i}\right)=\nu_{i}$ and $q\left({\tilde{\nu }}_{i}^{\prime }\right)=\nu_{i}^{\prime }$ for each *i*, we have

$$0+\overset{i-1}{\underset{j=1}{\sum }}\left(q\left({\tilde{\nu }}_{j}^{\prime }-{\tilde{\nu }}_{j}\right)\right)\in S_{N_{0}}\subseteq S_{N}.$$ (C2)

Hence, (C1) and (C2) imply that if *N*_*k*_ ≥ *N*_0_, then $X_{N_{k}}$ can re-enter $S_{N_{k}}$ from **0** with positive probability. Consequently, we constructed a sequence of reactions along which $X_{N_{k}}$ can re-enter $S_{N_{k}}$ from **x**_*k*_ for any *k* such that *N*_*k*_ ≥ *N*_0_. Since each $S_{N_{k}}$ is a communication class, $x_{k}\in S_{N_{k}}$, and in turn, this contradicts to the assumption that $x_{k}\in S_{N_{k}}$. Hence, the claim holds so that $S_{N}$ is closed for any *N* large enough.□

## APPENDIX D: PROOFS FOR LEMMA VI.1 AND THEOREM VI.2

Proof of Lemma VI.1. In the deterministic model $\tilde{x}\left(t\right)={\left({\tilde{x}}_{1}\left(t\right),\ldots ,{\tilde{x}}_{d}\left(t\right),y_{1}\left(t\right),\ldots ,y_{m}\left(t\right)\right)}^{⊤}$ associated with $\left(\tilde{S},\tilde{C},\tilde{R},\Lambda^{N}\right)$, if we fix *y*_*i*_(*t*) ≡ 1 for all *i* = 1, 2, …, *m*, then $\tilde{x}\left(t\right)$ follows the same ODE system as the deterministic model **x**(*t*) associated with the original network (S,C,R,Λ) does. Therefore, $\tilde{c}={\left(c^{*},1,1,\ldots ,1\right)}^{⊤}$ is a complex balanced steady state for $\tilde{x}\left(t\right)$. It has been shown that the existence of a single positive complex balanced steady state implies that all other positive steady states are complex balanced,^31^ therefore completing the proof.□

Proof of Theorem VI.2. Let **w**_*i*_ and *N*_*i*_ be the conservation vector and the conservation quantity of the slack network $\left(\tilde{S},\tilde{C},\tilde{R},\Lambda^{N}\right)$, respectively. Then, *π* is a stationary solution of the chemical master equation (3) of *X*,

$$\underset{\nu \to \nu^{\prime }\in R}{\sum }\lambda_{\nu \to \nu^{\prime }}\left(\;x-\nu^{\prime }+\nu \;\right)\pi \left(\;x-\nu^{\prime }+\nu \;\right)=\underset{\nu \to \nu^{\prime }\in R}{\sum }\lambda_{\nu \to \nu^{\prime }}\left(\;x\;\right)\pi \left(\;x\;\right).$$

Especially, as shown in Theorem 6.4, Ref*.* 32, *π* satisfies the *stochastic complex balance* for *X*: for each complex $\nu^{*}\in R$ and a state **x**,

$$\underset{\begin{array}{c} \nu \to \nu^{\prime }\in R\\ \nu^{\prime }=\nu^{*} \end{array}}{\sum }\lambda_{\nu \to \nu^{\prime }}\left(x-\nu^{\prime }+\nu \right)\pi \left(x-\nu^{\prime }+\nu \right)=\underset{\begin{array}{c} \nu \to \nu^{\prime }\in R\\ \nu =\nu^{*} \end{array}}{\sum }\lambda_{\nu \to \nu^{\prime }}\left(x\right)\pi \left(x\right).$$ (D1)

Let *q* be the projection function defined at (B1). Note that each reaction $\tilde{\nu }\to {\tilde{\nu }}^{\prime }\in \tilde{R}$ is defined as it satisfies the conservation law

$$w_{i}\cdot \left(\nu^{\prime }-\nu \right)+{\tilde{\nu }}_{d+i}^{\prime }-{\tilde{\nu }}_{d+i}=0,$$ (D2)

where $q\left(\tilde{\nu }\right)=\nu$ and $q\left({\tilde{\nu }}^{\prime }\right)=\nu^{\prime }$. Then, *π* also satisfies the stochastic complex balance for **X**^*N*^ because (D1) and (D2) imply that for each complex ${\tilde{\nu }}^{*}\in \tilde{R}$ and a state **x**,

$$\begin{array}{ll} & \underset{\begin{array}{c} \tilde{\nu }\to {\tilde{\nu }}^{\prime }\in \tilde{R}\\ {\tilde{\nu }}^{\prime }={\tilde{\nu }}^{*} \end{array}}{\sum }\lambda_{\tilde{\nu }\to {\tilde{\nu }}^{\prime }}^{N}\left(x-q\left(\nu^{\prime }-\nu \right)\right)\pi \left(x-q\left(\nu^{\prime }-\nu \right)\right)\\ & \;=\underset{\begin{array}{c} \nu \to \nu^{\prime }\in R\\ {\tilde{\nu }}^{\prime }={\tilde{\nu }}^{*} \end{array}}{\sum }\lambda_{\tilde{\nu }\to {\tilde{\nu }}^{\prime }}\left(x-\nu^{\prime }+\nu \right)\pi \left(x-\nu^{\prime }+\nu \right)\overset{r}{\underset{i=1}{\prod }}1_{\left\{N_{i}-w_{i}\cdot \left(x-\nu^{\prime }+\nu \right)\geq {\tilde{\nu }}_{d+i}\right\}}\\ & \;=\underset{\begin{array}{c} \nu \to \nu^{\prime }\in R\\ {\tilde{\nu }}^{\prime }={\tilde{\nu }}^{*} \end{array}}{\sum }\lambda_{\tilde{\nu }\to {\tilde{\nu }}^{\prime }}\left(x-\nu^{\prime }+\nu \right)\pi \left(x-\nu^{\prime }+\nu \right)\overset{r}{\underset{i=1}{\prod }}1_{\left\{N_{i}-w_{i}\cdot x\geq {\tilde{\nu }}_{d+i}^{*}\right\}}\\ & \;=\underset{\begin{array}{c} \nu \to \nu^{\prime }\in R\\ \nu =\nu^{*} \end{array}}{\sum }\lambda_{\nu \to \nu^{\prime }}\left(x\right)\pi \left(x\right)1_{\left\{N_{i}-w_{i}\cdot x\geq {\tilde{\nu }}_{d+i}^{*}\right\}}\\ & \;=\underset{\begin{array}{c} \tilde{\nu }\to {\tilde{\nu }}^{\prime }\in \tilde{R}\\ \tilde{\nu }={\tilde{\nu }}^{*} \end{array}}{\sum }\lambda_{\tilde{\nu }\to {\tilde{\nu }}^{\prime }}^{N}\left(x\right)\pi \left(x\right). \end{array}$$

Then, by summing up for each ${\tilde{\nu }}^{*}\in \tilde{R}$,

$$\begin{array}{ll} & \underset{\tilde{\nu }\to {\tilde{\nu }}^{\prime };\in \tilde{R}}{\sum }\lambda_{\tilde{\nu }\to {\tilde{\nu }}^{\prime }}^{N}\left(x-q\left(\nu^{\prime }-\nu \right)\right)\pi \left(x-q\left(\nu^{\prime }-\nu \right)\right)\\ & \;=\underset{\tilde{\nu }\to {\tilde{\nu }}^{\prime }\in \tilde{R}}{\sum }\lambda_{\tilde{\nu }\to {\tilde{\nu }}^{\prime }}^{N}\left(x\right)\pi \left(x\right), \end{array}$$

and in turn, *π*_*N*_ is a stationary solution of the chemical master equation of **X**^*N*^. Since the state space of **X** and **X**^*N*^ differ, *M*_*N*_ is a constant such that the sum of *M*_*N*_*π*(**x**) over the state space of **X**^*N*^ is one. Then, *π*_*N*_ = *M*_*N*_*π*.

## APPENDIX E: LYAPUNOV FUNCTIONS FOR EXAMPLE SYSTEMS

### 1. Lotka–Volterra with migration

We construct a Lyapunov function satisfying the conditions of Theorem V.4 for the Lotka–Volterra model with migration [Fig. 4(a)]. First, for both the original model and the slack network, we assume *A*(0) = *a*_0_, *B*(0) = *b*_0_ that do not depend on *N*.

Let *V*(**x**) = *e*^*w*·**x**^ with *w* = (1, 1)^*⊤*^. The condition in Theorem V.4 holds. Note that the slack network of Fig. 4(a) admits a conservation law *A* + *B* + *Y* = *N* and the state space of the system $S_{N}$ is {(*a*, *b*)|*a* + *b* ≤ *N*}. Then, condition 2 in Theorem V.4 also follows by the definition of *V*.

Now, to show condition 3 in Theorem V.4, we first note that for **x** = (*a*, *b*),

$$\begin{array}{ll} & \underset{\nu \to \nu^{\prime }\in R}{\sum }\lambda_{\nu \to \nu^{\prime }}\left(x\right)\left(V\left(x+\nu^{\prime }-\nu \right)-V\left(x\right)\right)\\ & \;=e^{w\cdot x}\underset{\nu \to \nu^{\prime }\in R}{\sum }\lambda_{\nu \to \nu^{\prime }}\left(x\right)\left(e^{w\cdot \left(\nu^{\prime }-\nu \right)}-1\right)\\ & \;=V\left(x\right)\left(\kappa_{1}b\left(e^{-1}-1\right)+\kappa_{2}\left(e-1\right)+\kappa_{3}\left(e^{-1}-1\right)\right.\\ & \;+\left.\kappa_{4}a\left(e^{-1}-1\right)+\kappa_{5}a\left(e-1\right)\right). \end{array}$$ (E1)

Since we assumed $\frac{\kappa_{4}}{\kappa_{5}}=3$ (see the caption of Fig. 4), each coefficient of *a* and *b* is negative. Thus, there exists *M* > 0 such that for each **x** ∈ {(*a*, *b*) | *a* > *M* or *b* ≥ *M*}, the left-hand side of (E1) satisfies

$$\underset{\nu \to \nu^{\prime }\in R}{\sum }\lambda_{\nu \to \nu^{\prime }}\left(x\right)\left(V\left(x+\nu^{\prime }-\nu \right)-V\left(x\right)\right)\leq -CV\left(x\right)$$

for some *C* > 0. Letting

$$D=\left(C+1\right)\underset{a\leq M\text{and}b\leq M}{\max}\underset{\nu \to \nu^{\prime }\in R}{\sum }\lambda_{\nu \to \nu^{\prime }}\left(x\right)\left(V\left(x+\nu^{\prime }-\nu \right)-V\left(x\right)\right),$$

condition 3 in Theorem V.4 holds with *C* and *D*.

### 2. Protein synthesis with a slow toggle switch

We also make use of the Lyapunov approach shown in Theorem V.4 to prove that the first passage time of the slack system in Fig. 5(b) converges to the original first passage time as the truncation size *N* goes to infinity. Let $X^{N}=\left(X,Z,D_{0}^{X},D_{1}^{X},D_{0}^{Z},D_{1}^{Z}\right)$ be the stochastic system associated with the slack system. Recall that the slack network admits a conservation relation **w** · **X**^*N*^ ≤ *N* with **w** = (1, 1, 0, 0, 0, 0). Hence, we define a Lyapunov function *V*(**x**) = *e*^*x*+*z*^, where **x** = (*x*, *z*, *dx*_0_, *dx*_1_, *dz*_0_, *dz*_1_). By the definition of *V*, it is obvious that condition 1 in Theorem V.4 holds. Furthermore, since $S_{N}=\left\{x\;|\;x+z\leq N\right\}$, condition 2 in Theorem V.4 also holds. So, we show that condition 3 in Theorem holds.

For a reaction $\nu \to \nu^{\prime }\in R_{I}≔\left\{Z+D_{0}^{X}\to D_{1}^{X},X\to 0,X+D_{0}^{Z}\to D_{1}^{Z},Z\to 0\right\}$, it is clear that the term *V*(**x** + *ν*′ − *ν*) − *V*(**x**) is negative. For each reaction *ν* → *ν*′ in $R_{I}^{c}$, it is also clear that the term *V*(**x** + *ν*′ − *ν*) − *V*(**x**) is positive. However, the reaction intensity for a reaction in $R_{I}$ is linear in either *x* or *z*, while the reaction intensity for a reaction in $R_{I}^{c}$ is constant. Therefore, there exists a constant *M* > 0 such that if *x* > *M* or *z* > *M*, then

$$\begin{array}{ll} & \underset{\nu \to \nu^{\prime }\in R}{\sum }\lambda_{\nu \to \nu^{\prime }}\left(x\right)\left(V\left(x+\nu^{\prime }-\nu \right)-V\left(x\right)\right)\\ & \;\leq C^{\prime }\underset{\nu \to \nu^{\prime }\in R_{I}}{\sum }\lambda_{\nu \to \nu^{\prime }}\left(x\right)\left(V\left(x+\nu^{\prime }-\nu \right)-V\left(x\right)\right)\\ & \;=-CV\left(x\right) \end{array}$$

for some constants *C*′ > 0 and *C* > 0. Hence, letting

$$D=\left(C+1\right)\underset{x\leq M\text{and}z\leq M}{\max}\underset{\nu \to \nu^{\prime }\in R}{\sum }\lambda_{\nu \to \nu^{\prime }}\left(x\right)\left(V\left(x+\nu^{\prime }-\nu \right)-V\left(x\right)\right),$$

condition 3 in Theorem V.4 holds with *C* and *D*.

### 3. The exclusive mutual inhibition, self-activation model

We show that *V*(**x**) = *e*^*x*+*z*^ is a Lyapunov function satisfying the conditions in V.4 for each **x** in the state space $S=\left\{x=\left(x,z,a00,a01,a10,b00,b01,b10\right)\in Z_{\geq 0}^{8}\;|\;a00+a01+a10=1,b00+b01+b10=1\right\}$of ExMISA model (22). Note that every reaction producing either *X* or *Z* in (22) has a constant order reaction intensity because the counts of genes *A* and *B* cannot exceed 1. On the contrary, each reaction consuming either *X* or *Z* has either linear or quadratic reaction intensity. Let $R_{I}$ be a collection of reactions in the ExMISA model that degrade either *X* or *Z* such as *A*_00_ + 2*X* → *A*_01_. Then, in the same way we showed in Appendix E 2, there exists a constant *M* > 0 such that for each x∈S, if *x* > *M* or *z* > *M*, then

$$\begin{array}{ll} & \underset{\nu \to \nu^{\prime }\in R}{\sum }\lambda_{\nu \to \nu^{\prime }}\left(x\right)\left(V\left(x+\nu^{\prime }-\nu \right)-V\left(x\right)\right)\\ & \;\leq C^{\prime }\underset{\nu \to \nu^{\prime }\in R_{I}}{\sum }\lambda_{\nu \to \nu^{\prime }}\left(x\right)\left(V\left(x+\nu^{\prime }-\nu \right)-V\left(x\right)\right)\\ & \;=-CV\left(x\right) \end{array}$$

for some constant *C*′ > 0 and *C* > 0. Hence, letting

$$D=\left(C+1\right)\underset{x\leq M\text{and}z\leq M}{\max}\underset{\nu \to \nu^{\prime }\in R}{\sum }\lambda_{\nu \to \nu^{\prime }}\left(x\right)\left(V\left(x+\nu^{\prime }-\nu \right)-V\left(x\right)\right),$$

condition 3 in Theorem V.4 holds with *C* and *D*. Finally, note that $S_{N}=\left\{x\;|\;x+z\leq N\right\}$ since the slack system admits a conservation bound

$$w\cdot x\leq N\text{ with}w={\left(1,1,0,0,0,0,0,0\right)}^{⊤}.$$

Hence, conditions 1 and 2 in Theorem V.4 hold.
